# Water Peel-Off Transfer of Electronically Enhanced, Paper-Based Laser-Induced Graphene for Wearable Electronics

**DOI:** 10.1021/acsnano.2c07596

**Published:** 2022-11-16

**Authors:** Tomás Pinheiro, Ricardo Correia, Maria Morais, João Coelho, Elvira Fortunato, M. Goreti F. Sales, Ana C. Marques, Rodrigo Martins

**Affiliations:** † CENIMAT|i3N, Departamento de Ciência de Materiais, Faculdade de Ciências e Tecnologia, Universidade Nova de Lisboa and CEMOP/UNINOVA, Campus da Caparica, 2829-516Caparica, Portugal; ‡ BioMark@UC, Department of Chemical Engineering, Faculty of Science and Technology, Coimbra University, 3030-790, Coimbra, Portugal; § CEB − Centre of Biological Engineering, University of Minho, 4710-057, Braga, Portugal

**Keywords:** laser-induced graphene, paper, transfer methodologies, wearable electronics, electrochemical sensors, strain sensors, microsupercapacitors

## Abstract

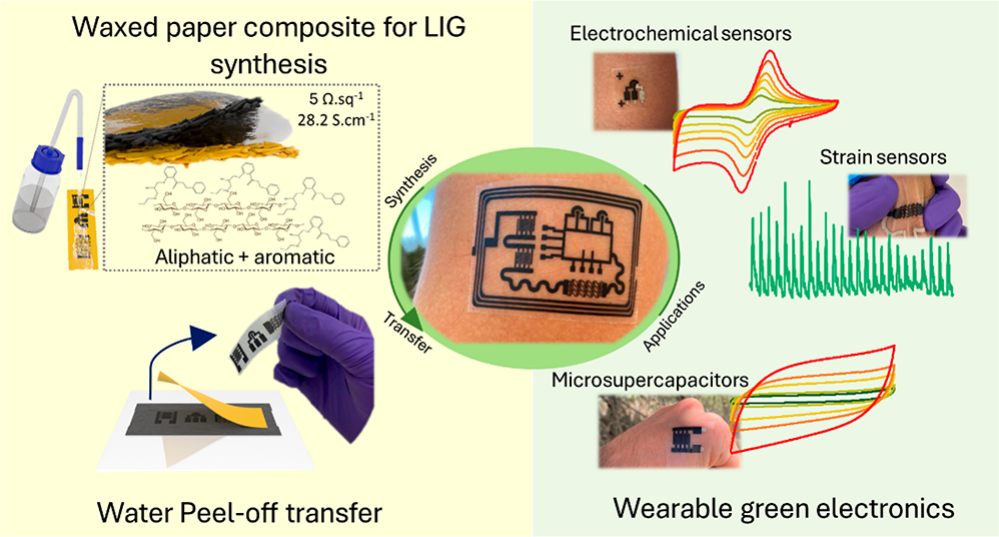

Laser-induced graphene (LIG) has gained preponderance in recent years, as a very attractive material for the fabrication and patterning of graphitic structures and electrodes, for multiple applications in electronics. Typically, polymeric substrates, such as polyimide, have been used as precursor materials, but other organic, more sustainable, and accessible precursor materials have emerged as viable alternatives, including cellulose substrates. However, these substrates have lacked the conductive and chemical properties achieved by conventional LIG precursor substrates and have not been translated into fully flexible, wearable scenarios. In this work, we expand the conductive properties of paper-based LIG, by boosting the graphitization potential of paper, through the introduction of external aromatic moieties and meticulous control of laser fluence. Colored wax printing over the paper substrates introduces aromatic chemical structures, allowing for the synthesis of LIG chemical structures with sheet resistances as low as 5 Ω·sq^–1^, translating to an apparent conductivity as high as 28.2 S·cm^–1^. Regarding chemical properties, *I*_D_/*I*_G_ ratios of 0.28 showcase low defect densities of LIG chemical structures and improve on previous reports on paper-based LIG, where sheet resistance has been limited to values around 30 Ω·sq^–1^, with more defect dense and less crystalline chemical structures. With these improved properties, a simple transfer methodology was developed, based on a water-induced peel-off process that efficiently separates patterned LIG structures from the native paper substrates to conformable, flexible substrates, harnessing the multifunctional capabilities of LIG toward multiple applications in wearable electronics. Proof-of concept electrodes for electrochemical sensors, strain sensors, and in-plane microsupercapacitors were patterned, transferred, and characterized, using paper as a high-value LIG precursor for multiples scenarios in wearable technologies, for improved sustainability and accessibility of such applications.

Ever since its discovery, laser-induced graphene (LIG) has established itself as a very attractive material for electrode fabrication. For LIG, the straightforward fabrication stands on the need for minimal infrastructure and precursor materials, in a maskless, catalyst-free, nontoxic synthesis route, allowing for low-cost, high-throughput patterning of graphenic structures with very high selectivity and localized conversion, within fully customizable geometries.^1^ This brings advantages when comparing to conventional graphene synthesis methods, such as graphite exfoliation, chemical vapor deposition (CVD), or crystal epitaxy, that require expensive and complex manufacturing equipment and processes.^2^ As such, LIG has found space in the production of planar microelectronic devices, due to the resulting porous nature of the three-dimensionally stacked 2D lattices of the converted graphene structures, that circumvent some cumbersome processes of single-layer graphene transfer, patterning, and stacking for the production of functional architectures.^3,4^ As a consequence, LIG has been used in many applications, ranging from energy harvesting and storage devices, such as triboelectric nanogenerators (TENGs) and microsupercapacitors (MSCs),^5^ photovoltaics,^6^ electrophysiological signal monitoring,^7^ biophysical sensors and actuator systems,^8^ and electrochemical sensing.^9^ Most of these applications have been developed on plastic polymers, mainly polyimide (PI), resulting in LIG with very attractive intrinsic properties, such as high specific areas (around 340 m^2^/g), good thermal stability (>900 °C), and attractive electrical properties (5–25 S/cm).^10^ More recently, other precursor substrates have been put forward to improve on the accessibility and environmental impact of such plastic substrates.^11^ These include wood,^12^ leaves,^13^ and other organic biomass such as cork.^14^ Besides these, other more refined forms of extracted precursor materials have been used, including aromatic-rich lignin composite films^15^ and cellulose-rich substrates, such as cardboard^16^ and paper sheets.^17^ Natural organic substrates can achieve very good electrical properties, with sheet resistances as low as 10 Ω·sq^–1^, contingent on the presence of aromatic precursors, namely, lignin, that can serve as a template for graphitization.^12^ However, aliphatic-rich cellulosic substrates, such as paper, lack these electrical properties, since they do not possess the intrinsic aromatic carbon structures that aid in this graphitization process. With cellulose being the most abundant polymer in the world, its inclusion in fabrication chains in electronics is of interest, including as a high-value LIG precursor.

Another important aspect of LIG is its application in flexible microelectronic elements. This is empowered by the native flexible properties of the used plastic polymers or organic substrates, which lead to bound LIG microstructures that retain the mechanical properties of the selected native substrate. As such, many applications have been developed, targeting flexible, wearable electronics and bioelectronics.^18^ However, these native substrates have some shortcomings when envisioning stretchable, fully conformable applications, due to low elasticity of commercial forms of these materials.^19,20^ Hence, many authors have developed different transfer techniques, to move LIG microstructures into elastomeric polymers, that can add this improved flexibility and conformability. These transfer techniques can be inspired by Scotch-tape exfoliation of graphite, where adhesives are used to anchor LIG architectures to the transfer substrate.^21,22^ Alternative approaches have been presented, by casting elastomer polymers over the scribed LIG structures, to produce elastomeric films that can anchor the bulk LIG volume. Such methods have been translated to use with a range of elastomers, including polydimethylsiloxane (PDMS) and other silicone elastomers,^7,23^ poly(methyl methacrylate) (PMMA),^24^ Ecoflex,^25^ and substrates with other properties, such as biodegradable starch films,^26^ concrete, or epoxy resins.^27^ Another fabrication of stretchable LIG microelectronic components has been the embedding of LIG precursors into elastomeric films, which are then submitted to laser irradiation and patterning. Such approach has been applied for the fabrication of MSCs in PDMS embedded with PI^28^ and pressure sensors from lignin-embedded PDMS.^29^ Using these methodologies, very attractive electronic elements can be fabricated; however, presently, none of these approaches have been adapted to the use of more accessible substrates such as paper and other cellulosic materials. This may be attributed to some technical drawbacks. Paper has high adhesion to adhesive glues, because they can incorporate its porous structure, inhibiting a direct peel-off. The same is observed for casting of elastomeric polymers over paper, ultimately hindering the peel-off without ripping the fibrous paper structure.

In this work, we expand the conductive properties of paper-based LIG, through a set of modifications that improve the conversion efficiency of cellulosic substrate into conductive graphenic structures, paired with meticulous control of laser operational parameters. Furthermore, a simple transfer method is developed, to harness these enhanced capabilities into flexible, conformable substrates, targeting applications for wearable electronics. The modifications imposed on the paper substrate are based on the introduction of aromatic carbon chemical structures, which promote a more efficient rearrangement of cleaved carbon bonds in the laser irradiation process. The imposed treatment on the paper substrates is divided into two stages, first by the introduction of fire-retardant chemicals, which increase the thermal resistance of the cellulose fibers, hindering their decomposition and ablation. Boron-based chemicals, such as borax, suffer endothermic decomposition and release additional bound water molecules, acting as chemical heat sinks,^30,31^ which help dissipate the very high localized temperatures upon laser irradiation. Second, the paper substrates are treated with colored paraffin wax, to introduce aromatic compounds that can serve as templates for more efficient reorganization and intramolecular condensation upon the buildup of graphene lattices within the synthesized LIG. As previously shown for other substrates, such as wood, the amount of aromatic components, in this case lignin, is paramount to obtain improved chemical and conductive properties in the synthesized LIG structures.^12^ As such, the control over the amount of colored wax used to modify the paper substrates is a simple way to control the efficiency of the laser induction and tailor the conductive properties of LIG patterns. In addition, a thorough control of the laser fluence applied to the substrate was employed, to study and optimize the outcomes upon the conversion process, by manipulating key variables including laser source power, lasing scan speed, focus of the laser beam, and the number of lasing scans, used to increase the graphitization efficiency.^32,33^ With such control over the conversion of wax-modified paper substrates, the conductive and chemical properties reached in this work are on par with LIG synthesized using aromatic-rich plastic polymers, such as PI and organic materials such as wood. Single-digit sheet resistance, low defect density, and high degree of crystallinity of the obtained lattices are attainable with this modification, introducing paper into the toolbox of high-efficiency LIG precursor materials. With these enhanced properties, versatile, simple transfer methodologies for paper-based LIG patterns are of interest, to target more comprehensive applications in flexible and wearable technologies. In this case, patterned and transferred electrodes for electrochemical and strain sensors and in-plane microsupercapacitors were developed, showing the applicability and multifunctionality of this material, while improving on aspects of accessibility and cost reduction, within many sustainable production and fabrication frameworks, such as the United Nations Sustainable Development Goals or the European Union Green Deal.

## Results and Discussion

### Boosting the Properties of Paper-Based LIG through Colored Paraffin Treatment and Laser Fluence Control

**Figure 1:**
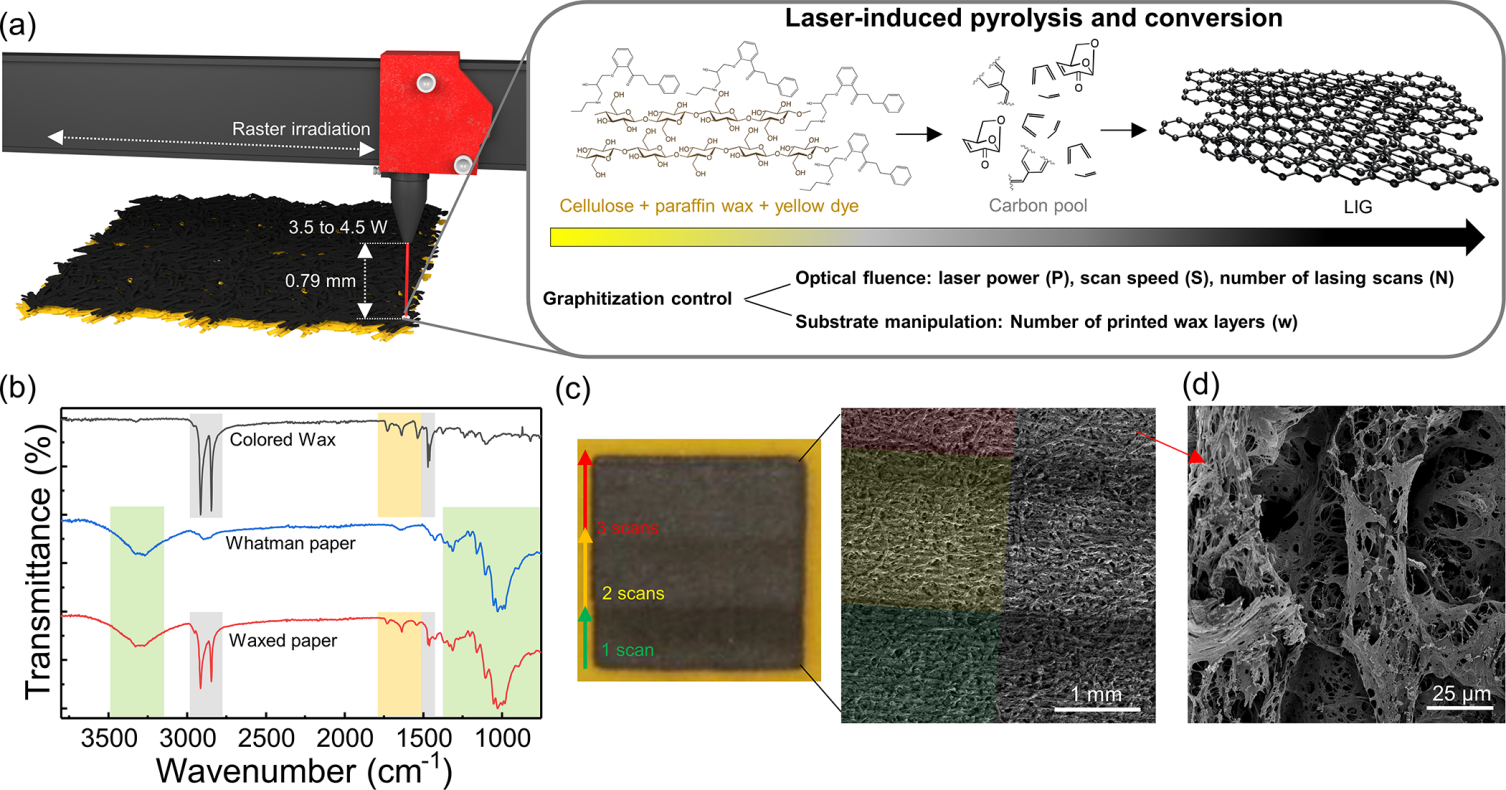
Synthesis of LIG from wax-modified chromatography paper. (a) Schematic representation of the conversion mechanism. (b) FTIR spectra of LIG precursor materials, showing the introduction of aromatic moieties into the volume of paper substrate from colored paraffin wax. (c) Optical image of converted LIG square with three distinct regions, corresponding to the number of lasing scans and the resulting SEM micrograph. (d) SEM micrographs of converted LIG after three lasing scans.

Paper substrates can be directly converted to LIG by direct laser writing (DLW), using commercial CO_2_ laser sources, after appropriate fire-retardant chemical treatments that increase their thermal resistance.^33^ The aliphatic carbon rings within the polymeric structure of cellulose are cleaved and reorganized into graphitic structures, with the degree of aromaticity and resulting graphenization dependent on the imposed optical fluence over the substrate. However, due to the absence of aromatic carbon chemical arrangements in its composition, which are more prone to graphenization,^12^ the efficiency of these laser conversion processes has been inferior when compared to other substrates, such as PI or wood, resulting in less attractive conductive and chemical properties of the synthesized, paper-based LIG. As such, the introduction of aromatic chemical structures is proposed as a means of improving the graphenization process upon DLW of paper substrates, paired with a thorough control of optical fluence, dictated by laser operational parameters. The mechanism for the synthesis of electrically enhanced paper-based LIG is schematically illustrated in Figure 1a. Using a 10.6 μm CO_2_ laser source with a 50 W maximum power, paraffin wax-treated paper substrates are irradiated under specific conditions, to achieve LIG patterns with single-digit sheet resistance (*R*_s_) and improved chemical properties, showcased by Raman profiles with improved defect densities. First, the modified paper substrates are irradiated with a specific distance from the laser beam exit nozzle of 0.79 mm, in order to control the laser spot size and energy density imposed over the substrate by unit of area. Using different spot size focus profiles has been shown to improve the efficiency of laser-induced conversion processes, by creating laser spot superposition patterns that act as if the same area is multiply lased, boosting the continuity of the patterned material within both a single rastered line and consecutive lines.^11^ Upon this irradiation, the aliphatic carbon rings within cellulose chemical structures and the hydrocarbon and aromatic structures present in the paraffin and in the yellow pigment used to color the paraffin wax suffer laser-induced pyrolysis, with photothermal cleavage of C–O–C, C–C, and C–O bonds, causing depolymerization, deoxygenation, and dehydration of the native chemical structures. This pyrolysis process creates a carbon pool that serves as a template for reorganization and aromatization, leading to the necessary graphitization of the substrate and LIG production, aided by the presence of increased amounts of native aromatic chemical structures. In this case, this commercial form of paraffin wax contains two benzene structures bound with straight chain hydrocarbons and is mixed with resin and yellow dye,^34^ which can also contain aromatic carbon rings within their chemical structures. Dyes and pigments usually possess conjugated systems where there are alternating double and single bonds within aromatic structures, which have resonance of electrons giving rise to absorption in specific wavelengths of the visible spectrum.^35^ The paper substrates and modified paper substrates were studied with Fourier transform infrared spectroscopy (FTIR), to identify the chemical bonds within their structure, presented in Figure 1b. As can be seen, some of the characteristic peaks for paraffin wax can be identified in the spectrum of this colored wax (highlighted in gray), namely, carbon–hydrogen stretching and bending bands from its −CH_3_ and −CH_2_ groups (719, 1463, 2849, and 2917 cm^–1^).^36^ Besides these peaks, there are different overtones, from 1500 to 1750 cm^–1^, and some of them could be attributed to C=C aromatic rings and their stretching,^37^ arising from the aromatic components in paraffin and the yellow dye contained in it (highlighted in yellow). Although the specific pigment used for this wax printing formulation is not disclosed by the manufacturer, a thorough survey of chemical structures associated with pigments and dyes shows the abundance of aromatic-rich chemistry in these components.^38^ When printing wax layers over the paper substrate, the resulting spectra contain all these components associated with colored paraffin wax, besides the ones associated with the aliphatic ring arrangements of the polymeric structure of cellulose, such as the peaks associated with C–O–C and C–O bonds around 1000 cm^–1^ (highlighted in green). As such, it is seen that the aromatic moieties contained in the paraffin wax are introduced in the volume of the paper substrate, with their density able to be controlled by the number of printing cycles (Figure S1). Furthermore, the use of more wax printing cycles leads to a more complete filling of paper porosity, turning the distribution of the modifying wax more uniform, until a maximum point reached at five wax layers (Figure S2).

To control the degree of graphitization of the modified substrate upon laser irradiation, the fluence applied to the substrates was manipulated, by imposing distinct laser powers, raster scan speeds, and number of patterning scans to the substrate, treated with varying layers of colored paraffin wax. In Figure 1c, a square patterned within a paper substrate treated with four wax layers and using 4 W laser power is presented, with three different regions patterned with a different number of lasing scans. In region 1, single lasing performed with a scan speed of 15.2 cm·s^–1^ is imposed, while in region 2, double lasing is imposed, maintaining the same scan speed for the first scan and decreasing the scan speed to 12.7 cm·s^–1^ for the second scan. In the third region, three lasing cycles are imposed, maintaining the same conditions of region 2 and performing the third scan with a scan speed of 10.2 cm·s^–1^. The rationale for decreasing the scan speed for subsequent lasing scans is based on the increased conversion efficiency observed for lower speeds, caused by an increased optical fluence.^39^ Thus, the first scan serves as a first carbonization step, with higher speed that minimizes damage to the substrate, followed by scans with lower scanning speeds, for increased cleavage and reorganization of bonds. The SEM micrograph of the patterned square is presented, showing marked differences between each region. With the increase in the number of lasing scans, the overall coloration of the LIG surface changes and tends to become more metalized, a first indication that the pattern may become more conductive. Furthermore, it is possible to observe the effect of raster scans over the substrate, with the expected horizontal line patterns, that do not destroy the native fibrous architecture of the paper substrate. In terms of the architecture of individual fibers after the irradiation process, presented in Figure 1d, it is possible to discern that additional porosity is imposed over the substrate, caused by decomposition of organic chemical structures of the treated paper substrate and consequent release of volatiles. Furthermore, it is visible that wax previously filling paper pores is removed after irradiation, indicating that improvements in conductivity are not imposed by the presence of additional conductive material, but by more efficient graphitization of cellulose fiber templates. In addition, irradiation of paper composites with distinct amounts of wax does not lead to significant structural and morphological changes, in terms of the fibrous nature of the material, besides slightly smoother fiber surfaces for higher printing cycles. No results are presented for unmodified paper as reference, since without the inclusion of the solid wax ink, the described irradiation regimens lead to complete ablation of paper, even with the presence of a fire retardant. This shows how the proposed modification scheme further increases the thermal resistance, allowing for the application of higher power regimens and resulting temperatures for carbonization and graphitization.

**Figure 2:**
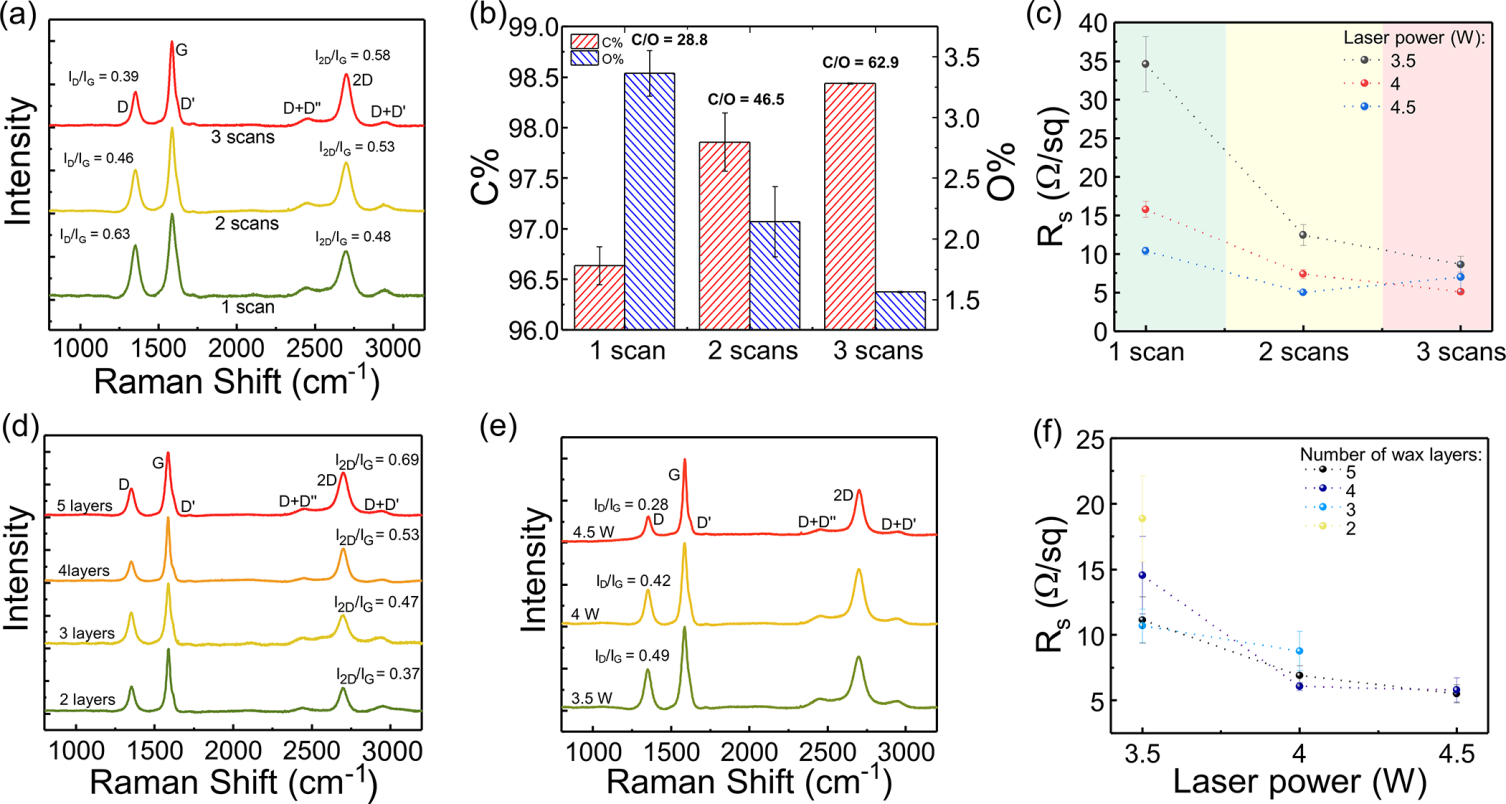
Characterization of LIG synthesized on wax-modified paper. (a) Raman spectra on the effect of the number of lasing scans used for conversion and (b) EDS relative elemental analysis of resulting LIG, using paper modified with four wax layers and 4 W laser power. (c) Sheet resistivity of LIG applying one to three lasing scans and varying laser powers over paper modified with four wax layers. (d) Raman spectra on the effect on the amount of wax used to modify paper substrates. (e) Raman spectra on the effect of increasing laser source power on LIG chemical properties. (f) Sheet resistivity measurements on the effect of increasing laser source power for LIG synthesized using three lasing scans and paper modified with varying wax layers (2 to 5).

When it comes to the resulting chemical and conductive properties of LIG synthesized using these conditions, characterization was performed using Raman spectroscopy, energy dispersive X-ray spectroscopy (EDS), X-ray photoelectron spectroscopy (XPS), and four-point probe electrical measurements for *R*_s_ assessment. Figure 2a presents normalized Raman spectra of LIG patterned on paper modified with four wax layers, 4 W imposed power, and varying number of lasing scans. From the analysis of the spectra, it is possible to see that the synthesized LIG has a low defect density, characterized by low-intensity D peaks, and good crystallinity, characterized by intense 2D peaks. Furthermore, a comparison of the spectra shows how the use of multiple lasing scans is a simple strategy to improve the chemical properties of LIG. In this case, both *I*_D_/*I*_G_ and *I*_2D_/*I*_G_ ratios indicate an improvement in these properties when more lasing scans are imposed. For a single scan approach, the *I*_D_/*I*_G_ has a value of 0.63, decreasing to a value of 0.39 for triple lasing scan. Contrarily, the *I*_2D_/*I*_G_ ratio increases from 0.48 to 0.58, showing a more crystalline, ordered LIG. This improvement in the Raman profiles of LIG is paired with an increase in the superficial, relative carbon content of LIG films, demonstrated by EDS measurements presented in Figure 2b. For a single-scan patterning, a C/O ratio of 28.8 is achieved, tripling the relative carbon content for three lasing scans, with a C/O ratio of 62.9, showing a more efficient cleavage of undesired C–O bonds and their subsequent release as gases. To determine the effect of this improvement of chemical properties upon the conductive properties, *R*_s_ determination showed that the increase in lasing scans leads to a decrease of this parameter (Figure 2c). For a single lasing scan, the conversion process of paper treated with four wax layers leads to *R*_s_ values ranging from 34.6 Ω·sq^–1^ (10.3% RSD, *n* = 5) to 10.38 Ω·sq^–1^ (4.7% RSD). This shows the utility of waxed paper, which allows for the synthesis of LIG with *R*_s_ values on par with conventional polyimide. When the number of lasing scans is increased, *R*_s_ decreased for all the tested laser power values. For 3.5 W, triple lasing scan leads to an *R*_s_ value of 8.6 Ω·sq^–1^ (11.8% RSD), already achieving single-digit values. However, for increasing laser powers, it is possible to reduce this resistance, with *R*_s_ lowering to 5.1 Ω·sq^–1^ (7.3% RSD) for 4 W laser power and 5.0 Ω·sq^–1^ (3.8% RSD) for 4.5 W lasing power, using two lasing scans. The resulting *R*_s_ shows improved conductivities when compared to reports in the literature for several precursor materials, such as PI, which reached minimum values around 15 Ω·sq^–1^,^10^ wood, which presented an *R*_s_ around 10 Ω·sq^–1^,^12^ and other cellulosic substrates, which reached minimum values around 30 Ω·sq^–1^.^40^ For triple lasing scan using 4.5 W power, there is an excessive laser fluence, which causes degradation of the substrate and LIG chemical structures, leading to a slight increase in *R*_s_. Thus, lasing power was limited to 4.5 W for further investigations.

After showing how the use of wax-treated paper, paired with control of laser fluence, can result in improved chemical and electrical properties of LIG films, Raman analysis was employed to determine the effect of the amount of wax toward the graphitization process. In Figure 2d, Raman spectra of LIG films synthesized on papers treated with two to five wax layers, 4 W laser power, and three lasing scans are presented. The main finding when comparing the spectra is the improvement of the *I*_2D_/*I*_G_ ratio, which evolves from a value of 0.37 (11.0% RSD, *n* = 6) for two wax layers to 0.69 (2.4% RSD) when five layers are employed. This shows that for the same lasing conditions the presence of increasing amounts of wax leads to more crystalline, ordered LIG chemical structures, since there is a higher density of aromatic chemical structures that boost the graphitization potential of the substrate, by acting as nucleation centers where aromatization and buildup of graphene lattices can start and progress toward highly graphitic structures. Taking the most crystalline, ordered condition of five wax layers, the effect of applied lasing power was surveyed, showing similar effects to the increase in the number of lasing scans. For progressively higher laser power (Figure 2e), the high degree of crystallinity is paired with an improvement on defect density, showing how laser fluence influences the rearrangement of carbon bonds toward more pristine 2D lattices composing the 3D LIG films. For a lasing power of 3.5 W, an *I*_D_/*I*_G_ ratio of 0.49 (11.9% RSD) was reached, improving to 0.28 (22.6% RSD) for 4.5 W. The fingerprints of these chemical properties, associated with these combinations, are reflected on the resulting *R*_s_ measurements for conditions employing three lasing scans, presented in Figure 2f. The tendency of the conductive properties of LIG patterns is to improve with the increase in the number of printed wax layers and lasing power employed for conversion. When fewer wax layers are applied, *R*_s_ values are higher and the substrate suffers excessive degradation, not being conducive for conductivity measurements. For increasing number of wax layers, higher lasing powers can be employed for conversion, since there is more material to be pyrolyzed, ultimately improving the thermal resistance of the substrate and the resulting graphitization outcomes. Using these strategies, single-digit *R*_s_ values can be consistently achieved, with multiple combinations reaching values around 5 Ω·sq^–1^. Overall, this simple wax treatment of paper substrates toward improved chemical and conductive properties reaches the best values of Raman peak ratios and *R*_s_ of any report using cellulosic substrates, to the best of our knowledge, showing potential for application in the development of electrodes and components for planar microelectronics. In addition, multiple experimental conditions using wax-treated paper result in very attractive chemical and conductive properties.

### Water Peel-Off Transfer of Paper-Based LIG

**Figure 3:**
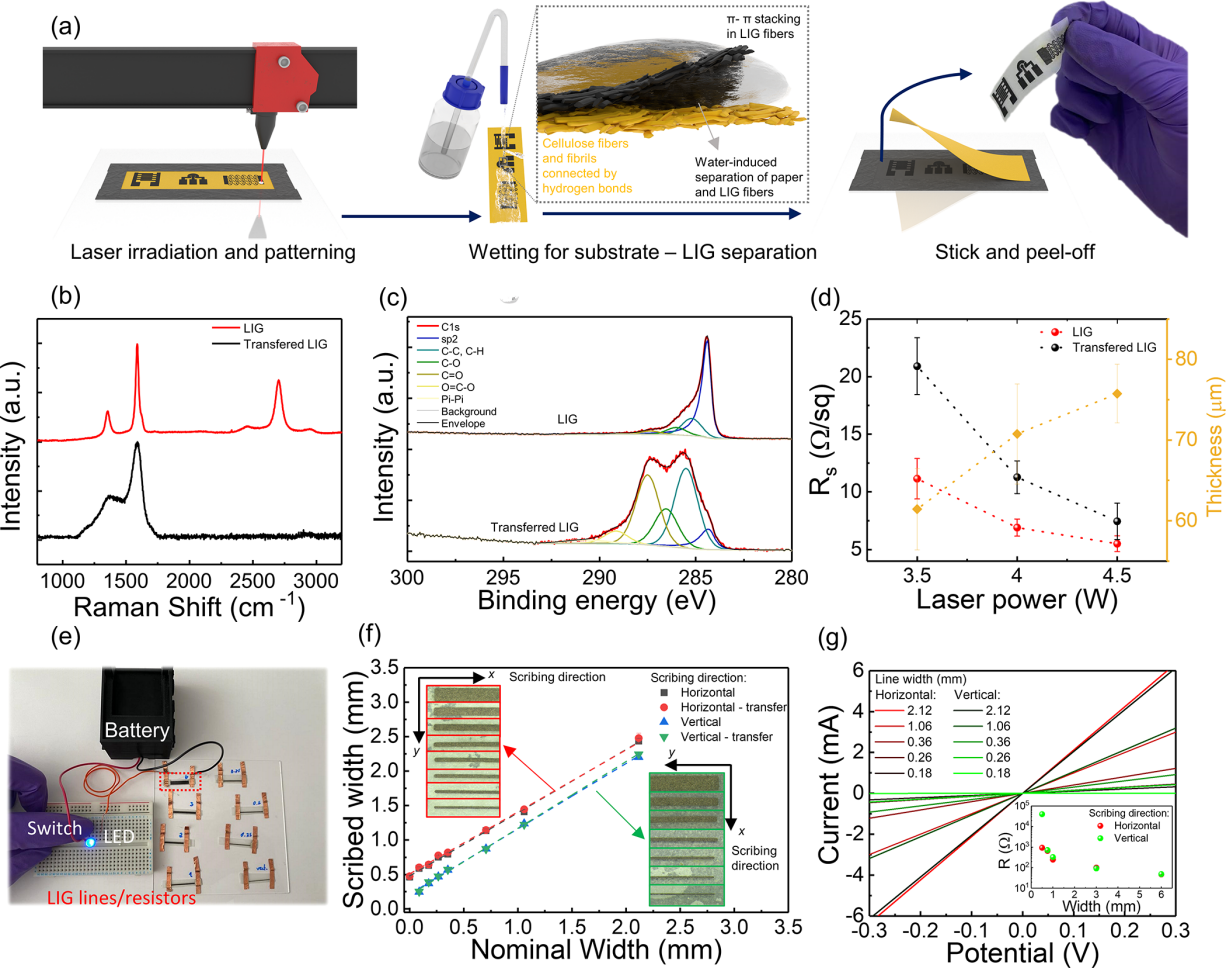
Water-induced peel-off transfer process of paper-based LIG and characterization of transferred LIG chemical structures. (a) Schematic representation of the three-step transfer process, starting with laser scribing and patterning of functional architectures, wetting of substrate and LIG patterns for mild separation, and sticking and peel-off of LIG patterns onto the transfer substrate. (b) Raman and (c) XPS C 1s deconvoluted spectra of pristine and transferred LIG. (d) Sheet resistivity of pristine and transferred LIG. (e) Transferred LIG electric lines and resistances at an electric circuit for connection of a battery and LED. (f) Analysis of nominal vs scribed line widths before and after transfer, depending on scribing direction. (g) *I*–*V* curves and resistance of transferred lines, depending on scribing direction.

After enhancing the capability of paper substrates to be high-value LIG precursors, through the introduction of aromatic moieties that boost the graphitization of the modified substrate, its applicability toward flexible, conformable electronic elements was studied. In Figure 3a, a schematic representation of the developed transfer methodology is presented. Starting with the irradiation and patterning of any desired LIG structure, this transfer method can be applied to a versatile range of geometries targeting different applications, from interdigitated electrodes to planar electrochemical cells and other electrode formats. After the patterning of any desired functional LIG design, the paper sheet is removed from its glass support and is subjected to a wetting step. This has the purpose of mildly separating converted LIG chemical structures from any native cellulose fibers and fibrils not affected by the irradiation. Cellulose fibers and fibrils present a much higher affinity toward water, due to the abundance of hydrogen bonds, which are important at cellulose’s intermolecular, fibril, and fiber levels.^41^ Contrarily, the converted material and its interlayer interactions are mostly dominated by π–π stacking of converted graphene layers and weak van der Waals forces,^42^ creating a defined interface between native and converted material. Upon the wetting process, water interacts with cellulose fibers at the interfacial level, creating additional hydrogen bonds, which promote their separation from LIG chemical structures. Following this step, the wetted paper substrate can be applied to any desired transfer substrate, with the requirement that it possesses adhesive properties over the transfer surface. Thus, a simple stick and peel-off process is necessary for the complete transfer of LIG functional patterns, where water acts both to separate LIG–cellulose phases and to prevent the adhesion of paper to the adhesive transfer layer, due to the abundant hydrogen bonds formed between water and cellulose, without the need for any specific pressure requirements for the release of LIG onto the transfer substrate. As a transfer substrate model, a medical grade polyurethane tape with a polyacrylate glue surface was selected (Leukoplast Fixomull), due to its elastic properties, ideal to characterize transferred LIG structures in different scenarios (Supplementary Video 1). However, this transfer method can be applied to a myriad of substrates, as seen in Figure S3, contingent on the presence of an adhesive layer, which can be added to any desired transfer substrate, using repositionable spray adhesives, as was performed for transfer into PDMS.

To characterize the chemical and conductive properties of transferred LIG, Raman spectroscopy, XPS, and four-point probe measurements were performed. In Figure 3b, a comparison is drawn between LIG synthesized using 4.5 W power and three lasing scans, over a paper substrate modified with five wax layers. As can be seen, the expected Raman profile of pristine LIG previously presented is achieved. However, upon the transfer of the LIG structures, the downward surface of the pattern is exposed to the excitation laser, leading to a much different Raman profile. This downward surface is mostly constituted by more amorphous carbon forms, represented by the absence of the 2D peak and the increased width of G and D peaks. This evidence was confirmed by XPS (Figure 3c), used to survey the C 1s spectra of pristine and transferred LIG. For a pristine LIG surface, the deconvolution of the C 1s spectra shows the preponderance of the peak associated with sp^2^ carbon bonds, characteristic of synthesized graphitic structures. In addition, there is a low density of different C–O bonds, characteristic of the native materials, showing a high conversion efficiency. In opposition, the surface exposed after transfer shows less density of sp^2^ carbon and higher preponderance of sp^3^ carbon bonds and remaining C–O, C=O, and O–C=O bonds, more abundant at the native wax-printed cellulose substrate. This shows that the photothermal phenomenon governing the conversion of the substrate to LIG, upon exposure to the CO_2_ laser beam, is less efficient with the increase in conversion depth, leading to less pristine graphitic structures over higher depths and more abundance of oxygen moieties. Furthermore, it can be hypothesized that considering the tubular nature of cellulose fiber architectures, fiber surfaces directly exposed to the laser beam will be more efficiently converted, while the opposing surfaces are less exposed to the high temperatures imposed over the irradiation process. This influences not only the microscopic effects over individual fibers but also the macroscopic properties of the surface exposed after transfer. This is slightly reflected in the measured *R*_s_ values for transferred and pristine LIG patterns (Figure 3d). When comparing the values after transfer, an increase in the resistive properties of patterns is observed, mainly for lower applied laser power. For 3.5 W, *R*_s_ increases to 20.9 Ω·sq^–1^ (11.8% RSD, *n* = 5), almost doubling the resistance. For 4 W, this difference is not as significant, with *R*_s_ values of 11.2 Ω·sq^–1^ (12.6% RSD), and for 4.5 W the difference is less significant, with patterns achieving values of 7.5 Ω·sq^–1^ (11.5% RSD), showing that the same levels of single-digit *R*_s_ can still be reached for transferred LIG. However, due to the increase in amorphous carbon contents at the exposed surface after transfer, the real sheet resistance of LIG may not be reflected, because these carbon forms are the ones in contact with the measurement probes, leading to an increase in resistivity. This is also an important consideration for applications where the surface chemistry and conductivity of LIG is a key parameter. In conjunction with sheet resistance, pattern thickness was analyzed by cross-section measures (Figure S4). For increasingly higher power regimens, pattern thickness increases, as expected by the higher temperatures that effect cellulosic structures at higher depths (Figure 3d). Taking into consideration these thickness values, estimation of conductivity shows the improved conductive properties when compared to other reports of paper-based LIG (Table S1). For LIG bound to paper and synthesized using 4.5 W, a mean conductivity of 24.3 S·cm^–1^ (11.1% RSD, *n* = 5) was reached, reaching values as high as 28.2 S·cm^–1^. For transferred LIG patterns using the same condition, a mean conductivity of 19.3 S·cm^–1^ was reached. Comparing these metrics with other LIG forms, this paper-based LIG reaches the best conductivity levels reported for PI (25–34 S·cm^–1^),^10^ wood (23.3 S·cm^–1^),^43^ and other engineered lignocellulosic precursors (28 S·cm^–1^),^44^ while far exceeding previous reports for paper-based LIG, as evidenced in Figure S5. Considering the porosity of paper, effective conductivity values that consider the fibrous architectures of paper can be estimated. Using the Reynolds and Hugh rule,^44,45^ the effective conductivity of the carbon LIG material can be estimated by normalizing the volume fraction of carbon. Taking the porosity of Whatman grade 1 paper (48%)^46^ and not considering additional porosity caused by the irradiation process and volatile release, the effective conductivity of this carbon material can reach values as high as 67.3 S·cm^–1^.

To study the versatility of patterning and design of LIG structures and their transfer, the resolution and resistivity of LIG lines with varying widths were surveyed, to understand the influence of the directional dependency of the raster process over the conductive properties and the achieved width after irradiation, in opposition to the nominal width set in the laser control software. As can be seen in Figure 3e, these LIG lines can act as circuit elements, where the control of geometry dictates their resistive properties, allowing the flow of current in a simple circuit capable of turning an LED on and off. Lines with varying nominal widths were constructed, to determine the resulting scribed width. Depending on raster direction, the laser spot pulse density can be subjected to variations, which influence the resolution of the scribing process of any pattern, which is important in different functional designs that contain both horizontal and vertical elements within their geometry. For horizontally scribed lines, their length was scribed from left to right, following the raster movement of the laser mirror, with their width being set by consecutive raster cycles. Contrarily, for vertically scribed lines, their length was scribed from top to bottom, with the horizontal raster movement setting the width of the line. Results are presented in Figure 3f, showing a similar behavior of the scribed width for both the horizontal and vertical irradiation, with a linear variation associated with the increase in scribed width. However, it is visible that the resolution of the scribed lines varies with the scribing orientation. Vertically scribed lines have widths closer to the nominal width, while horizontally scribed lines present higher resulting widths, evidenced by the upward shift. Furthermore, it is only possible to pattern the minimum width set in the laser control software, corresponding to a width equal to the laser spot size, using a horizontal orientation. This is a result of the variation in pulse resolution of the used laser system, where for the *x* direction, it is set by the points per inch (PPI) setting of the laser systems, kept at 1000 for all the experiments. For the same set width, the number of raster cycles in the *y* direction is much lower than the PPI setting, resulting in these differences. For a horizontal scribing direction, the lowest achieved width was 464.4 μm, while for vertical scribing, the lowest width value was 252.1 μm, with no changes after line transfer, for both raster regimens. These results were complemented with the measurement of *I*–*V* curves for patterned lines, presented in Figure 3g. The results indicate that for higher line widths the difference in the measured resistance for horizontal and vertical scribing is not significant, with lines of 2 mm width achieving resistance values of around 50 Ω. With the decrease in patterned width, the resistance of the lines increases, with resistance of patterns in three different orders of magnitude, from 10^1^ Ω to 10^3^ Ω. Due to the variation in pulse density upon the irradiation process, there is a threshold at which the difference in resistance between vertical and horizontal scribing increases substantially, as is the case for a nominal width of 180 μm, where for horizontal scribing, the line presents a resistance of around 1 kΩ, while for vertical scribing, the resistance increases to approximately 40 kΩ. These are important considerations when miniaturizing LIG functional patterns, where a careful manipulation of operational parameters is crucial to achieve fine patterns that can approximate the maximum laser resolution, close to the laser spot size (127 μm at focal point). To test the reproducibility of line production and transfer, 200 lines (20 × 2.2 mm, 5 wax layers, 3 scans, and 4.5 W power) were fabricated and tested in terms of their conductive properties. For a first group of 100 lines bound to the paper substrates, a mean resistance of 53.9 Ω (9.6% RSD, *n* = 100) was reached, translating to a conductivity of 23.0 S·cm^–1^. For the second group, made of 100 transferred lines, a mean resistance of 62.4 Ω (12.8% RSD) was reached, translating to a conductivity of 19.9 S·cm^–1^. Furthermore, a yield of 93% was reached for the transfer group, considering unsuccessful transfer lines with resistance above 2.5 standard deviations. These conductivity results, estimated by line resistance and geometry, corroborate the conductivity assessed by sheet resistance measures, confirming the good conductivity of this paper-based LIG.

### Demonstration of Flexible, Wearable Electronics from Transferred Paper-Based LIG

**Figure 4:**
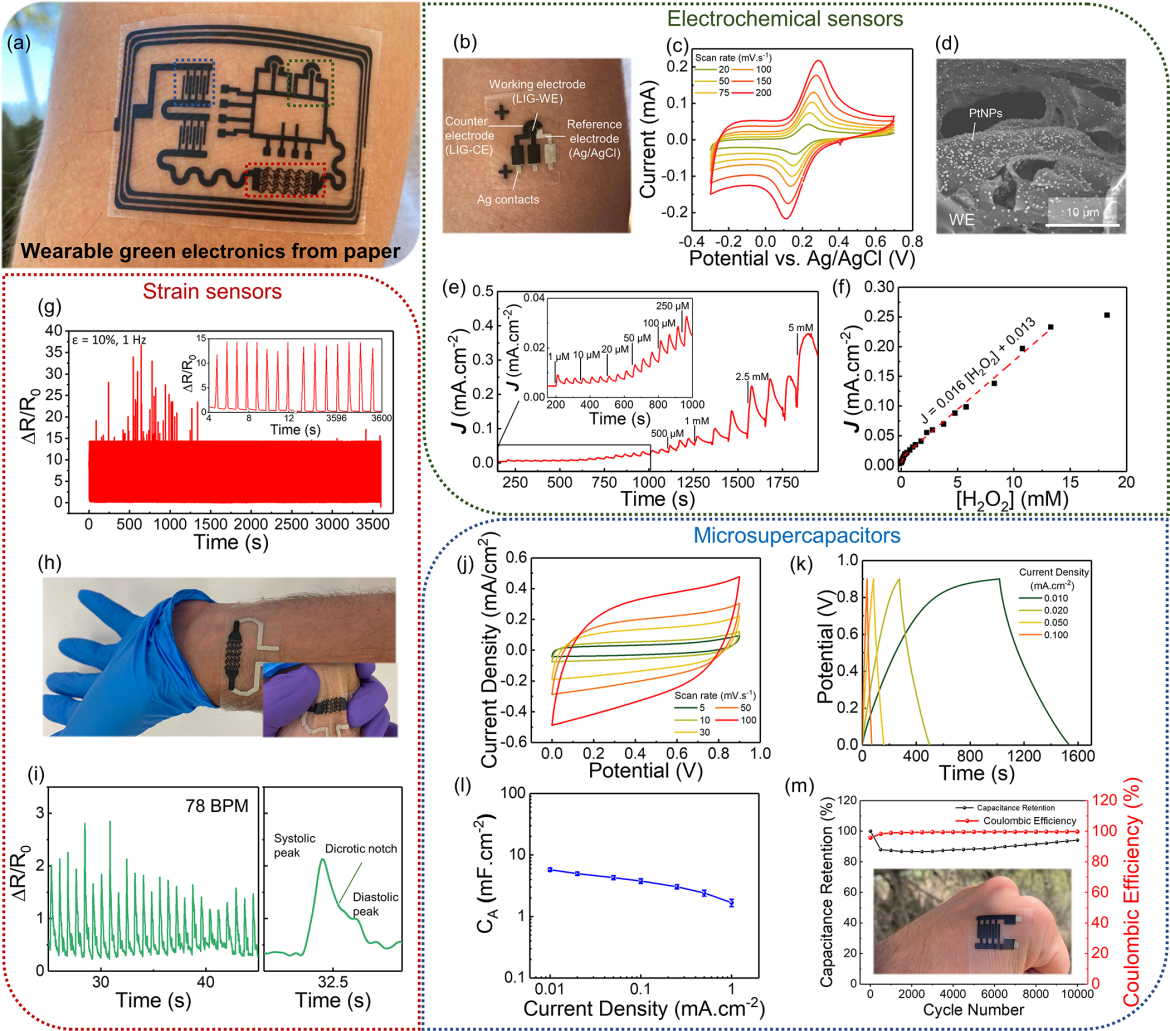
Demonstration of flexible, wearable applications for transferred paper-based LIG. (a) Illustrative circuit with planar microelectronic elements for skin-worn functional patches. (b–f) Flexible, skin-worn electrochemical planar cell development and characterization. (b) Electrode components for the developed cells on the skin. (c) CVs for Fe(CN)_6_^3–/4–^ redox probes at different scan rates (20–200 mV·s^–1^). (d) LIG-WE surface after PtNP electrodeposition. (e) Continuous CA response of PtNPs modified LIG-WE toward H_2_O_2_ at 0.7 V applied potential. (f) Amperometric calibration of a H_2_O_2_ sensor. (g–i) Wearable strain sensor from transferred paper-based LIG. (g) Bending strain cycling at 10% applied strain with 1 Hz frequency and 3600 cycles. (h) Strain gauge applied on the wrist, for pulse wave monitoring from the radial artery, with inset showing skin conformability of the LIG–polyurethane sensor. (i) Pulse wave signal for heart rate determination and distinction of systolic and diastolic signal phases. (j–m) Flexible, wearable MSCs from transferred paper-based LIG. (j) CVs of in-plane MSCs for different scan rates (5–100 mV·s^–1^). (k) Galvanostatic charge–discharge curves at different current densities (0.01–0.1 mA.cm^–2^). (l) Plot of areal specific capacitance vs current density. (m) Plot of capacitive retention and Coulombic efficiency over 10 000 charge–discharge cycles.

After demonstrating the excellent and tunable conductive properties enabled by LIG synthesis on wax-modified paper and its transferred form, the same flexible, stretchable polyurethane medical grade tape was used as a model transfer substrate. Optimized synthesis conditions of five wax layers, 4.5 W power, and three lasing scans were employed to exemplary pattern, transfer, and assemble different electronic components for wearable electronic systems, as depicted in Figure 4a. This exemplary patterned, skin-worn patch shows that different and complex electrode and connector geometries can be transferred. However, the use of LIG as a contact material for circuit fabrication is only exemplary, as LIG does not reach the same transport properties in terms of conductivity of contact materials such as metallic conductors. The first targeted application is the development of flexible, three-electrode electrochemical cells for electrochemical sensing applications. In this case, LIG patterning of carbon-based working and counter electrodes (LIG-WE and LIG-CE) was performed, followed by their transfer and subsequent patterning of a Ag/AgCl reference electrode (RE) and silver contacts using laser cut masks (Figure 4b). Electrochemical properties of LIG-based WE and CE within planar cells were studied, using a standard redox probe composed of ferri/ferrocyanide ions, with cyclic voltammograms (CVs) showing the characteristic anodic and cathodic peaks related to the electron transfer in this quasi-reversible process (Figure 4c). Low peak potential separations were achieved, below 200 mV for all the tested scan rates, showing very good electron transfer capabilities of the electrode surface. Applying the Nicholson method^47,48^ for the determination of the heterogeneous electron transfer (HET) rate constant *k*_0_ (Figure S6), an estimated value of 1.03 × 10^–2^ cm·s^–1^ (23.9% RSD, *n* = 5) was reached, which improves on previous reports from paper-based LIG electrochemical sensors^49,50^ and is of the same order of magnitude for LIG synthesized on polymeric substrates.^51^ Another important aspect analyzed using the Randles–Sevcik equation was the electrochemical surface area (Figure S6), which reached a value of 13.7 mm^2^ (16.9% RSD), an increase compared to the 8 mm^2^ geometric area of the WE. This indicates that even with the difference in surface chemistry of transferred LIG patterns previously studied, the surface morphology and chemistry allow for an efficient filling of the porous structure by aqueous electrolytes and diffusion of redox species, for effective electron transfer. To complement this characterization, electrochemical impedance spectroscopy (EIS) was used to characterize these planar, flexible cells. Nyquist and Bode plots are presented (Figure S6), showing low solution resistances around 100 Ω and charge transfer resistances (*R*_CT_) of 70 Ω, also presenting low impedances, with a maximum of 1.5 kΩ for 0.1 Hz. Furthermore, the electrochemical cells were exposed to different bending regimens (Figure S7), showing that they can operate in different bending environments for application in wearable situations.

Taking advantage of the attractive properties of these cells, platinum nanoparticles (PtNPs) were electrodeposited, to serve as strong catalysts toward hydrogen peroxide (H_2_O_2_) decomposition, as one of the most common transduction mechanisms in various enzymatic and nonenzymatic sensing schemes used for electrochemical sensing of different metabolites in mobile, wearable sensing modalities.^52^ SEM images of electrodeposited nanoparticles can be seen in Figure 4d and Figure S8, showing particles with sizes in the hundreds of nanometers, with a uniform and dense distribution over the WE surface. Noteworthy is the higher abundance of particles at more interior LIG fibers, most likely at more conductive and less amorphous LIG areas, as shown previously in the characterization of transferred LIG. Comparative CVs and EIS of electrochemical cells using the selected redox probes, before and after electrodeposition, were performed (Figure S9), showing an increase in current and decrease in *R*_CT_, associated with the increase in surface area given by PtNPs and their good conductivity. Proof-of-concept H_2_O_2_ electrochemical sensors were characterized using CV and chronoamperometry (CA), to determine the response of PtNPs-modified WE toward the decomposition of H_2_O_2_. In Figure S10, CVs show the appearance of both anodic and cathodic currents associated with H_2_O_2_ oxidation and reduction, respectively. To calibrate this H_2_O_2_ sensor, anodic currents associated with oxidation of H_2_O_2_ at positive applied potentials were used, since these are the most common applied potentials when this transduction mechanism is employed for enzymatic sensing of metabolites.^53^ In Figure S11, different applied potentials were used to study the progressive current increase associated with the addition of increasing concentrations of H_2_O_2_. A 0.7 V bias was selected, and the sensor response at this applied potential is presented in Figure 4e. This sensor presents a large response range, from the tens of μM to mM concentrations, showing the expected rise of current after the addition of H_2_O_2_, followed by a decrease until a steady state current level, associated with analyte consumption at the electrode surface. This response was calibrated by taking the current 25 s after the peak associated with H_2_O_2_ spiking, presented in Figure 4f. The sensor presents a large linear variation range between 50 μM and 13.2 mM, with a sensitivity of 16 μA·mM^–1^·cm^–2^ and a limit of detection (LOD) of 11.6 μM (3σ/S), appropriate for application in the development of more complex, enzyme-based biosensing schemes for sweat metabolite detection.^53^ A comparison of several LIG-based H_2_O_2_ electrochemical sensors is presented in Table S2.

The second proof-of concept application for flexible, transferred LIG is its application for strain sensor fabrication toward physiological monitoring. LIG-based strain sensors have been shown to present very attractive performances in various substrates,^17,54^ due to the piezoresistive properties of the patterned material. Serpentine-shaped strain sensors^55^ were patterned on wax-modified paper and transferred to medical grade polyurethane tape, being submitted to bending cycles at 10% strain, for 1 h, at 1 Hz frequency, as shown in Figure 4g. The sensor showed great stability over 3600 cycles, maintaining the same stretching and releasing signal shape, as shown by the inset. At this strain, a high gauge factor of 128.9 was achieved, potentiated by both the improved conductivity of LIG and the high flexibility of the transfer substrate, when compared to the native paper substrate. A comparison on the performance of several LIG-based strain sensors in the literature is presented in Table S3. Another interesting aspect when testing these strain gauges at higher strain regimens was the appearance of a cyclic triboelectric phenomenon, arising from the polyurethane tape charging. This is visible from the spikes in Δ*R*/*R*_0_ in Figure 4g and in Figure S12, where strain cycling with 20% strain was performed. This is an evidence of the ability of LIG to be used as a charge-collecting layer in architectures for the development of triboelectric nanogenerators.^56,57^ As a proof-of-concept application of a low-force sensitivity signal for noninvasive, wearable monitoring, strain gauges were applied in the radial artery, for pressure wave and heartbeat frequency monitoring, as shown in Figure 4h. A very good sensitivity and signal-to-noise ratio were achieved, allowing for a clear monitoring of the pulse waveform, as shown in Figure 4i, able to distinguish the systolic and diastolic phases of the pulse wave signal. Another means of measuring cardiac signals is through electrophysiological signal acquisition. For this purpose, a direct contact between electrodes and skin is needed, where the skin–electrode impedance is an important consideration. LIG circular electrodes of 5 mm diameter were fabricated and used to determine the impedance in the forearm region. Dry and wet electrode configurations were tested (Figure S13a). For a direct contact between skin and the LIG electrode, a very high impedance in the MΩ range was reached. When a salt-free conductive gel was used to improve this contact, the impedance lowered around 3 orders of magnitude, to around 30 kΩ at 100 Hz, which is conducive for electrophysiological signal monitoring.^58^ This variation is mainly due to the fibrous, microstructured nature of the LIG surface, which lowers the contact points between the skin and the electrodes. Using the wet electrode configuration, electrocardiogram (ECG) recordings of driven right leg (DRL) lead II configuration were performed (Figure S13b), showing a good signal-to-noise ratio and a clear identification of ECG signal components.

As a final application, transferred LIG was patterned into interdigitated electrodes to develop MSCs for energy storage purposes. As previously described, transferred LIG into polyurethane films holds good electrical properties, which makes it suitable for both a current collector and active material in energy storage systems. Thus, interdigitated in-plane MSC electrodes were fabricated under the optimized LIG parameters, using polyvinyl alcohol (PVA)/H_2_SO_4_ as electrolyte, and silver ink was applied on the electrodes to create electrical contacts. The electrochemical and energy storage properties of the produced MSC were thoroughly characterized by CV and galvanostatic charge–discharge (GCD) experiments. As shown in Figure 4j, the produced MSC presents a quasi-rectangular CV curve shape in a wide range of scan rates (5 to 100 mV/s), which is characteristic of electric double-layer capacitors (EDLCs) and indicates good electrochemical stability.^59,60^ Corroborating these results, GCD curves demonstrate that transferred LIG MSCs exhibit electrochemical capacitive behavior with an almost ideal symmetric triangular shape and very low voltage drop (Figure 4k). The areal specific capacitance (*C*_A_) from the GCD curves was calculated to be 5.76 ± 0.35 mF/cm^2^ for a current density of 0.010 mA/cm^2^ (Figure 4l), which is comparable with other published works.^10,61^ Even increasing the current density to 0.100 mA/cm^2^, the devices still exhibited a *C*_A_ of 3.77 ± 0.31 mF/cm^2^, confirming their good capacitive behavior. The stability over time of the produced LIG MSC was evaluated over 10 000 cycles of charge–discharge at a current density of 0.5 mA/cm^2^ (Figure 4m). As usual, a capacity loss is observed for the first cycles, upon which the system recovers to a final charge retention of 95% of the initial capacitance, which reveals a low-capacity loss during long cycling. The Coulombic efficiency is kept at 99.7% after 10 000 cycles. Moreover, the produced devices exhibited an energy density of 0.42 μWh/cm^2^ with a power density of 2.91 μW/cm^2^. A comparison of several LIG-based MSCs and their performance is presented in Table S4. Furthermore, the devices were tested in different bending regimens and after strain cycling over 2000 cycles (Figure S14), showing their applicability in wearable scenarios.

## Conclusions

As demonstrated in this work, the full potential of paper as a LIG precursor material is still unexplored, and many versatile, simple modification strategies can increase the graphitization potential of this material for laser irradiation processes toward LIG synthesis. The introduction of aromatic moieties by colored wax printing over the aliphatic-rich cellulose substrate results in a highly efficient precursor composite material, enabled by the adaptability of paper substrates toward varied modification strategies, including wax printing. This modification, paired with the meticulous control of operational laser variables, resulted in improved conductive LIG structures, with very low sheet resistances around 5 Ω·sq^–1^, translating to apparent conductivities as high as 28.2 S·cm^–1^. Furthermore, very attractive crystallinity and low defect density were achieved, observed by Raman profiles of LIG, which showed *I*_D_/*I*_G_ ratios as low as 0.28 and *I*_2D_/*I*_G_ ratios as high as 0.69. Furthermore, paper-based LIG is still unexplored in the fabrication of fully flexible, conformable components for wearable applications. As such, the developed water peel-off transfer methodology allows for paper to be used as an applicable LIG precursor in the fabrication of on-skin, conformable systems for wearable technologies. This transfer methodology achieves an efficient and complete transfer of LIG patterns that retain their optimized, enhanced conductive and chemical properties. The multifunctionality of paper-based LIG and its transferred form was demonstrated toward the development of components for wearable systems, through the fabrication of flexible, on-skin electrochemical planar cells, strain gauges, and in-plane MSCs, which were tested in different scenarios. With these results, paper can be considered in the toolbox of printed electronics materials as a more effective LIG precursor. Although LIG still does not reach some of the very appealing properties of printing inks made of 2D materials, including graphene and MXenes, its further study and functionalization with such materials can further boost its properties and applicability, while future efforts for integration of such components and performance optimization can lead to more accessible, sustainable wearable electronic systems for different scenarios, such as health monitoring, kinetic energy harvesting and storage, or interactive motion tracking.

## Methods

### Materials

During this work, laboratory grade ultrapure Milli-Q water (conductivity <0.1 μS/cm) was used to prepare all solutions, unless otherwise specified. Sodium tetraborate decahydrate (Na_2_B_4_O_7_·10H_2_O), potassium chloride (KCl), chroroplatinic acid hexahydrate (H_2_PtCl_6_·6H_2_O), sulfuric acid (H_2_SO_4_), and 30 wt % hydrogen peroxide solution were purchased from Sigma. Potassium hexacyanoferrate(III) (K_3_[Fe(CN)_6_]) and potassium hexacyanoferrate(II) trihydrate (K_4_(Fe(CN)_6_)·3H_2_O) were purchased from Roth. All reagents were used as received, without further purification. Whatman chromatography paper grade 1 (Whatman International Ltd., Floram Park, NJ, USA) was used for laser irradiation and LIG formation. As transfer substrate models, Leukoplast Fixomull medical grade polyurethane tape, Hypafix polyester wound dressing tape, polydimethylsiloxane, and 3M Transpore polyester fixing tape were used.

### LIG Synthesis on Wax-Modified Substrates and Peel-Off Transfer

Paper sheets were cut into A6 size (105 × 148 mm) and were submitted to a boron-based fire-retardant chemical treatment, as previously reported.^49^ The paper sheets were dipped into a 0.1 M solution of sodium tetraborate solution for 20 min, followed by drying at room temperature. Following this treatment, paper sheets were submitted to wax printing, using a Xerox Colorqube printer, using commercial yellow wax printing cartridges compatible with the printer. These cartridges are composed of paraffin wax (CAS #8002-74-2) and a proprietary yellow pigment. To control the degree of paraffin and yellow pigment loaded into the paper sheets, the number of printing cycles was varied, from two to five. Each printing cycle consists of printing a superficial layer of wax, followed by heating of the substrate over a hot plate, to permeate the wax throughout the volume of the paper. The loading capacity of the selected paper substrate was limited to five layers, since a complete pore filling is achieved and, for higher cycle numbers, there is a leakage of wax ink outside the paper structure.

After substrate treatment and modification, the paper sheets were put over a glass substrate and fixed with adhesive tape, to secure a flat paper surface for irradiation. A CO_2_ Universal laser system with a 10.6 μm wavelength, 50 W maximum power, and 127 mm·s^–1^ maximum scan speed was used for substrate irradiation and LIG synthesis. Some laser operational parameters were fixed during this process. The PPI was set at 1000, and the distance between the substrate and laser beam exit was fixed at 0.79 mm. Besides these variables, the laser source power, scanning speed, and number of lasing scans were varied, to study the graphitization outcomes upon laser irradiation. In addition, the irradiation processes were performed under a nitrogen-rich atmosphere.

After irradiation and patterning of LIG, the paper substrate was removed from the glass slide support, and the transfer substrate was fixed to the same support. To transfer the irradiated patterns, the paper substrate is submitted to a wetting step, where both surfaces are fully submerged in water. Following this wetting step, the patterned paper surface is placed on top of the adhesive surface of the transfer substrate with slight pressure, for 10 s. After the complete area of the paper surface is put into contact with the adhesive surface, the paper substrate is peeled off, leaving behind the patterned paper-based LIG.

### LIG Characterization

SEM characterization was performed using a Hitachi Regulus SU8220 system. Raman spectroscopy was performed in a Renishaw inVia Reflex micro-Raman spectrometer equipped with an air-cooled CCD detector and a HeNe laser. The laser beam was focused through a 50× Olympus objective lens. Measurements were performed with a 532 nm laser with 10 s exposure time and 3 accumulations as measurement parameters, with a laser power of 16 mW. X-ray photoelectron spectroscopy was performed using a Kratos Axis Supra, equipped with monochromated Al Kα radiation (1486.6 eV). Chemical composition was also studied through EDS mounted in an SEM (Hitachi TM 3030Plus tabletop). Electrical sheet resistance was determined by Hall effect measurements in Van der Pauw geometry in a Biorad HL 5500 equipment at room temperature.

### Fabrication and Characterization of Electrochemical Sensors

Three-electrode electrochemical cells were constructed by patterning LIG-WE and LIG-CE elements over wax-modified paper substrates and their subsequent transfer into the desired substrate. After transfer of these two elements, a mask was cut over a glassine paper to manually pattern a Ag/AgCl RE and silver contacts. After patterning, the glassine paper was removed and the cell was encapsulated, leaving open areas for the electrodes and contacts. Electrochemical measurements were performed using a PalmSens 4.0 potentiostat (PalmSens compact electrochemical interfaces). Prior to characterization, an electrochemical pretreatment cleaning step was performed on the electrodes, CV scanning a potential window from −2 to 2 V at a scan rate of 100 mV/s, using the supporting electrolyte (0.1 mmol/L KCl). After pretreatment, electrochemical cells were rinsed with water and left to dry in air. CV assays were carried out with a potential window from −0.3 to 0.7 V, at scan rates from 10 to 200 mV/s. EIS was carried with a pulse of 5 mV in amplitude, 50 data points, over a frequency range from 10.000 to 0.1 Hz. The electrochemical measurements by both CV and EIS were performed using a redox probe solution of 5.0 mmol/L [Fe(CN)_6_]^3–^ and [Fe(CN)_6_]^4–^, prepared in the supporting electrolyte. For PtNP electrodeposition, the CV technique was employed, by scanning a potential window between −0.2 and 0.7 V at 50 mV·s^–1^ for 20 cycles, using a 2.5 mM platinum salt solution in 60 mM H_2_SO_4_. Chronoamperometry measurements were performed by immersing the PtNP-modified sensors in 200 μL of phosphate-buffered saline (PBS) buffer (0.1 M, pH 7.4) and subsequently removed, and 10 μL of H_2_O_2_ solution in PBS buffer was added, to make the desired concentration.

### Fabrication and Characterization of Strain Sensors

Serpentine-shaped patterns based on previous literature^55^ were patterned over wax-modified paper and subsequently transferred into a desired substrate. After transfer, silver tracks were patterned using a glassine paper mask, followed by encapsulation of the sensor, so that LIG is not in contact with the skin for physiological signal monitoring. Characterization of strain sensors was performed using a custom-built bending apparatus controlled by an Arduino, allowing for the control of the bending radius and resulting applied strain and bending frequency. To monitor the signal arising from bending stimulus and radial artery pulse, a PalmSens 4.0 potentiostat was used, operating in chronopotentiometry (CP) mode. For CP, a constant current of 10 μA was applied, to monitor the potential changes and compute the variations in pattern resistance toward the applied mechanical stimuli.

### Fabrication and Characterization of MSCs

Patterning of interdigitated electrodes was performed over wax-modified paper, followed by its transfer, silver contact patterning, and encapsulation, leaving an open area for electrolyte placement. A PVA/H_2_SO_4_ aqueous gel was used as a solid electrolyte. In a standard procedure, 1 g of PVA was dissolved in 10 mL of distilled water at 90 °C under vigorous stirring for 1 h. Then, 0.5 mL of 98% H_2_SO_4_ was added and stirred for another hour. After the electrolyte deposition, the assembled MSCs were allowed to dry overnight at room temperature. The produced LIG MSC was characterized using a BioLogic SP-50 potentiostat (BioLogic Sciences Instruments) by means of CV (5 mV/s to 10 V/s) and GCD (0.010 to 1 mA/cm^2^) experiments. According to eq 1, the specific areal capacitance, *C*_A_, was calculated from the charge–discharge curves of the produced MSC.
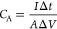
1where *I* is the applied current, Δ*t* is the discharge time, *A* is the MSC active area, and Δ*V = V*_2_ – *V*_1_ where *V*_2_ is the potential at the beginning of discharge and *V*_1_ is the potential at the end of discharge. The energy (*E*_A_) and power (*P*_A_) densities per unit area of the fabricated devices were calculated as follows:
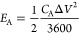
2
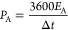
3where 3600 is a conversion factor from Ws to Wh.

## Supporting Information Available

FTIR spectra of wax-modified paper with different layers, SEM analysis of wax-modified paper with different layers and resulting LIG, transferred LIG patterns over various flexible substrates, optical cross-section analysis of transferred LIG, comparison table for chemical and conductive properties of LIG reports on various substrates, plot of state of the art of sheet resistance vs conductivity for LIG synthesized on various substrates, electrochemical characterization for electroactive area, heterogeneous electron transfer rate constant and electrochemical impedance spectroscopy of LIG electrochemical cells, characterization of electrochemical cells at different bending angles, SEM of electrodeposited PtNPs at a LIG working electrode, characterization of PtNP-modified cells, response of sensors to H_2_O_2_ by cyclic voltammetry, chronoamperometric analysis of H_2_O_2_ sensor response at various potentials, comparison table of LIG-based H_2_O_2_ sensors in the literature, strain sensor response at 20% strain, comparison table of LIG-based strain sensors in the literature, LIG electrode–skin impedance and ECG signal, characterization of MSCs at different bending angles, and comparison table of LIG-based MSCs in the literature (PDF)
Movie of transfer process and resulting wearable assembled onto skin (AVI)

## Supplementary Material

nn2c07596_si_001.pdf

nn2c07596_si_002.avi
