# Supplementary material for: Water Peel-Off Transfer of Electronically Enhanced, Paper-Based Laser-Induced Graphene for Wearable Electronics
Source: ACS Nano. 2022 Nov 16;16(12):20633–46. doi: 10.1021/acsnano.2c07596 (PMC9798867; doi:10.1021/acsnano.2c07596)
Supplement: Supplementary file 1 — nn2c07596_si_001.pdf [file nn2c07596_si_001.pdf]

Supporting Information

Water Peel-off Transfer of Electronically Enhanced, Paper-based Laser-induced Graphene for Wearable Electronics

Tomás Pinheiro [1,2,*], Ricardo Correia [1], Maria Morais, [1] João Coelho [1], Elvira Fortunato [1], M. Goreti F. Sales [2,3], Ana C. Marques [1,*], Rodrigo Martins [1,*]

[1] CENIMAT|i3N, Departamento de Ciência de Materiais, Faculdade de Ciências e Tecnologia, Universidade Nova de Lisboa and CEMOP/UNINOVA, Campus da Caparica, 2829-516 Caparica – Portugal

[2] BioMark@UC, Department of Chemical Engineering, Faculty of Science and Technology, Coimbra University, 3030-790, Coimbra, Portugal

[3] CEB – Centre of Biological Engineering, University of Minho, 4710-057, Braga, Portugal

[*] tp.pinheiro@campus.fct.unl.pt, accm@campus.fct.unl.pt, rfpm@fct.unl.pt

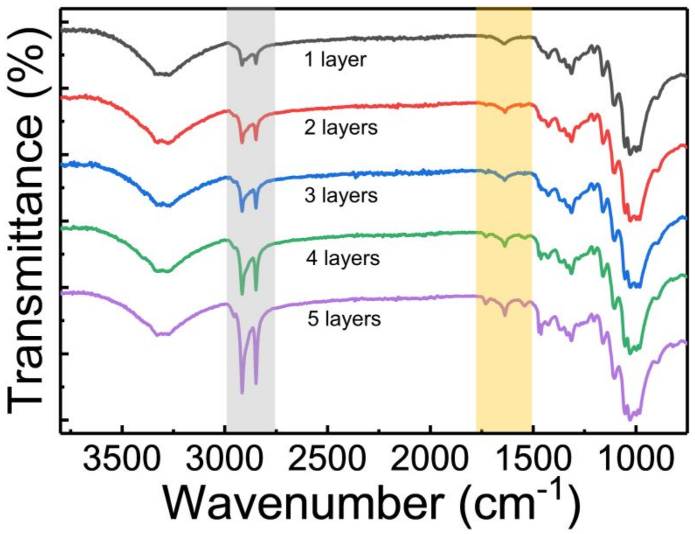

Figure S1 – FTIR spectra of chromatography paper substrates modified with increasing number of wax layers, showing the increase in intensity of peaks associated with both paraffin wax and yellow dye giving color to the printing wax

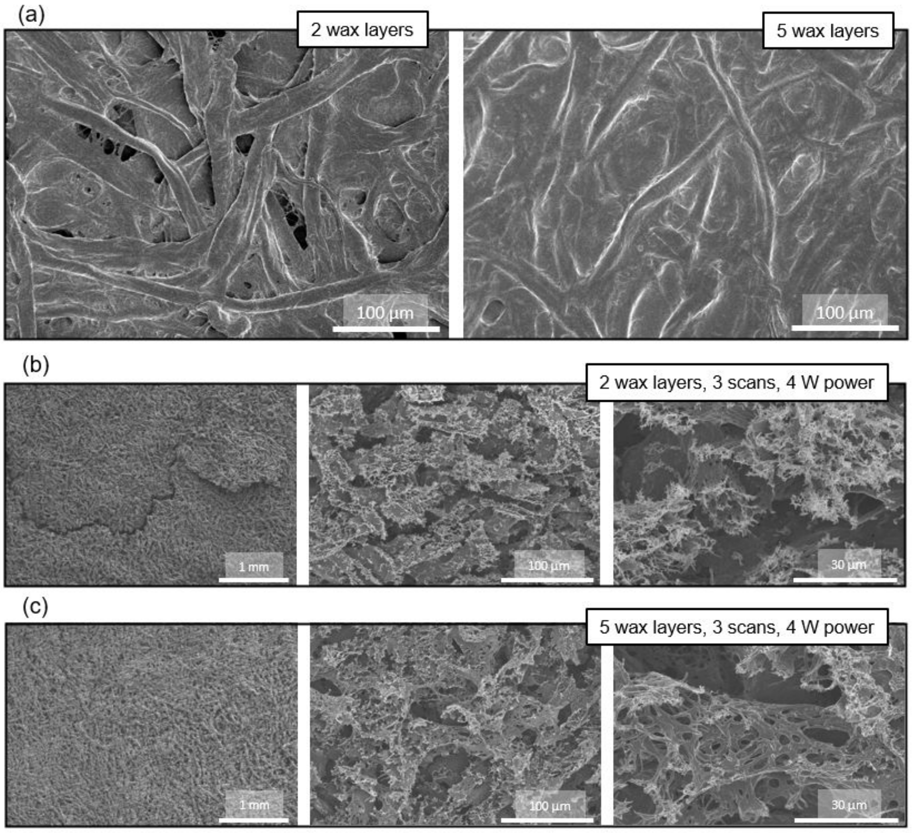

Figure S2 – SEM analysis of waxed paper and resulting LIG. (a) Paper-wax composite produced using 2 and 5 printing cycles. (b, c) LIG synthesized on wax-paper composite made with (b) 2 wax layers and (c) 5 wax layers.

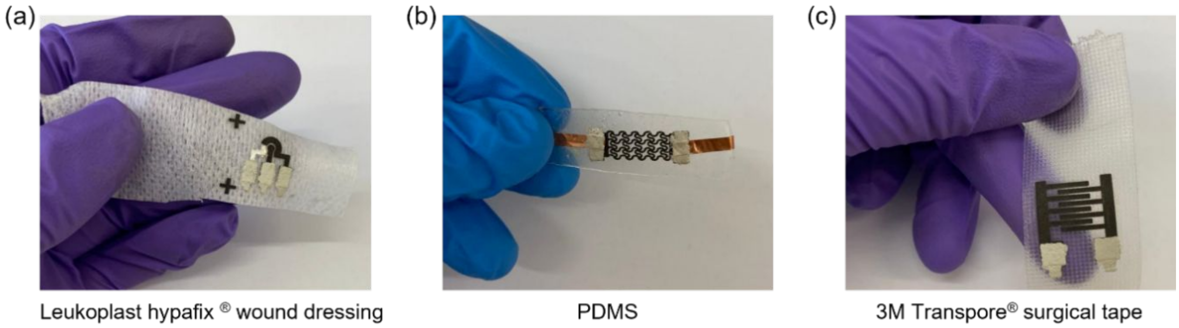

Figure S3 – Transferred LIG patterns over various substrates. (a) Transfer of electrodes for the fabrication of planar electrochemical cells on Leukoplast hypafix ® wound dressing. (B) Strain gauge transferred over PDMS, after spraying of a layer of repositionable adhesive for anchoring of LIG structures. (c) Transfer of interdigitated electrodes over 3M Transpore ® surgical tape containing a mild adhesive layer.

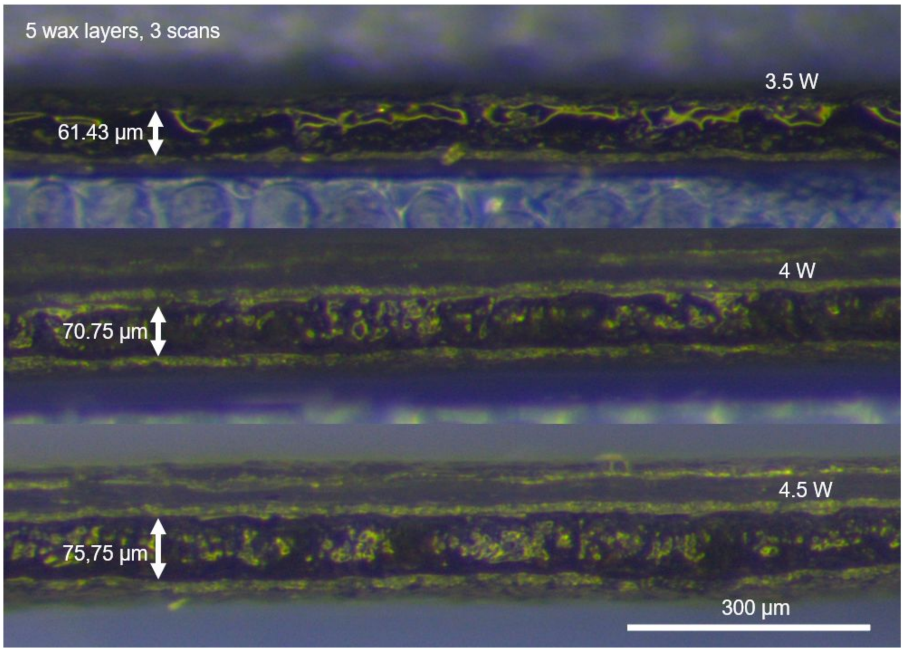

Figure S4 – Cross-section analysis of LIG squares used for sheet resistance measurements.

Table S1 – Comparison of Raman chemical features and conductivity of LIG from different precursors.

Precursor	Laser Source	I_d/I_g	I_{2d}/I_g	Sheet resistance ($\Omega.sq^{-1}$)	Conductivity ($S.cm^{-1}$)	Ref
PI	IR, CO_2	0.5	----	15	25	1
PI	IR, CO_2	1	1	36.9	34.4	2
PEI	IR, CO_2	0.2	0.65	15	6.7	3
Phenolic resin	IR, CO_2	----	----	40	4	4
Nomex paper	IR, CO_2	----	0.95	15	8.9	5
Wood	IR, CO_2	0.8	0.6	10	1.25	6
Wood	UV femtosecond laser (343 nm)	0.63	----	10	2.75	7
Wood with catalyst	IR, CO_2	0.25	0.75	20	25	8
Leaves	Yb-doped femtosecond fiber laser	----	----	23.3	4.8	9
Forest-based ink	IR, CO_2	----	----	3.8	28	10
Paper	IR, CO_2	0.88	0.84	61.5	0.95	11
Paper	IR, CO_2	0.5	----	32	2.94	12
Paper	IR, CO_2	0.6	1.3	56	5.95	13
Paper	IR, CO_2	----	----	100	1.17	14
Paper	Nd:YVO4 UV (355 nm)	----	----	132	2.0	15
Xylan	IR, CO_2	----	----	186	0.67	14
Wax-treated paper	IR, CO_2	0.28	0.69	5	28.2	This work

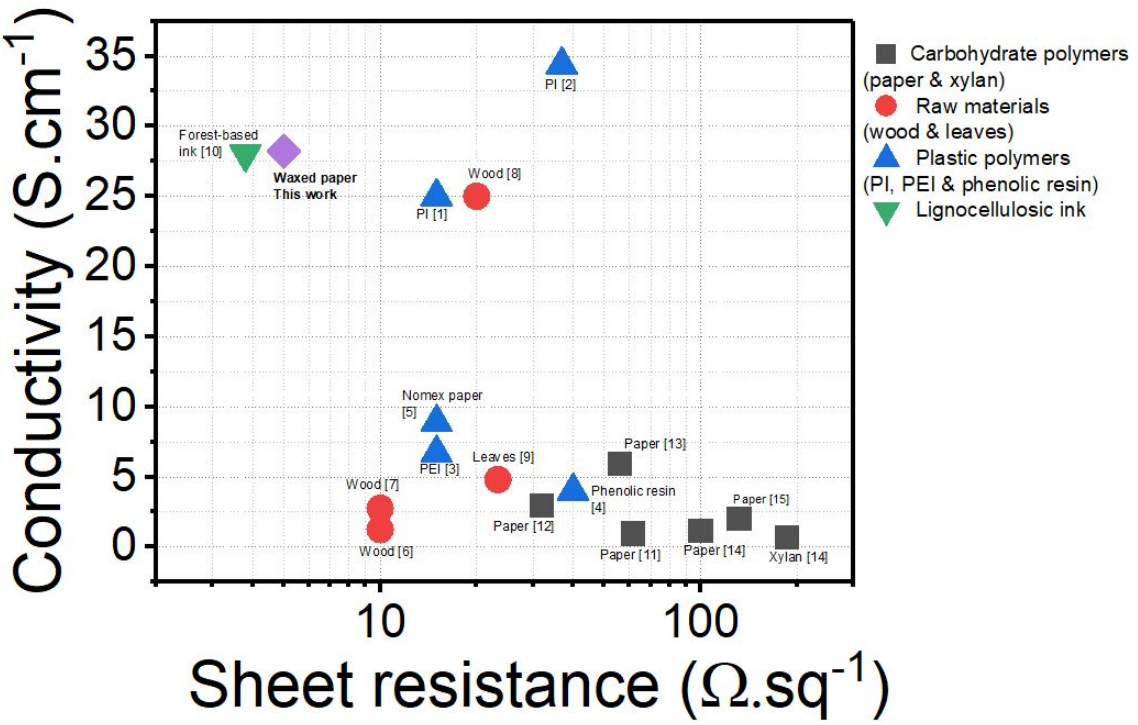

Figure S5 – Plot of sheet resistance vs. apparent conductivity of several LIG films produced from different precursor groups, including carbohydrate polymers, raw materials, plastic polymers and formulated inks.

Randles-Sevcik equation:

$$I_p^{quasi} = \pm 0.436 nFAC \sqrt{\frac{nFDv}{RT}}$$

Nicholson method for Heterogeneous Electron Transfer (HET) rate constant k_0:

$$\Psi = k^0 \sqrt{\frac{RT}{\pi FD\vartheta}}$$

or

$$\Psi = Ck^0 \vartheta^{-1/2}$$

$$\Psi = \frac{(-0.6288 + 0.0021\ X)}{(1 - 0.017\ X)}$$

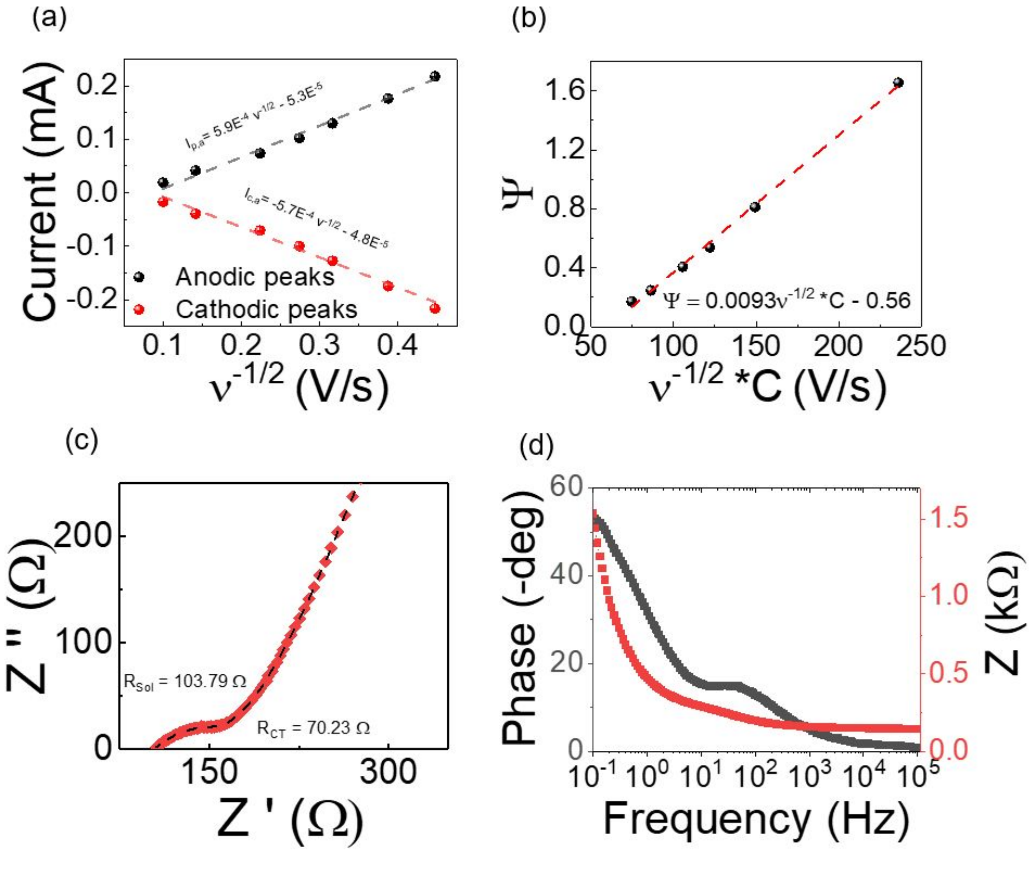

Figure S6 – Characterization of electrochemical planar cells. (a) Plot of anodic and cathodic peak currents *vs.* square root of scan rate, for calculation of electrochemically active area through the Randles Sevcik equation. (b) Plot of dimensionless kinetic parameter Ψ *vs.* square root of scan rate for the calculation of rate constant k_0. (C) Nyquist plot of an electrochemical cell for solution and charge transfer resistance determination. (D) Corresponding Bode plot.

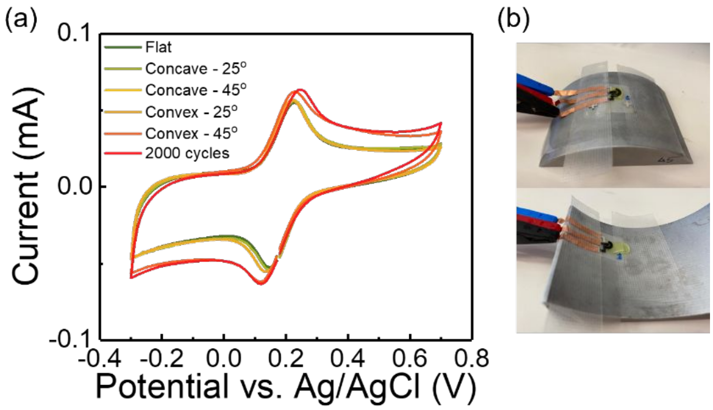

Figure S7– Characterization of electrochemical cells at different bending angles using CV at 50 mV.s^{-1}. (a) CVs showing small variation in peak position and current for concave (25 and 45°) and low convex (25°) angles and small increase in current and peak separation for high convex angle (45°) and after 2000 bending cycles with 5% strain, caused by a mild separation of LIG fibers and increase in the surface area of the LIG electrodes. (b) Image of electrochemical cells at concave and convex testing angles.

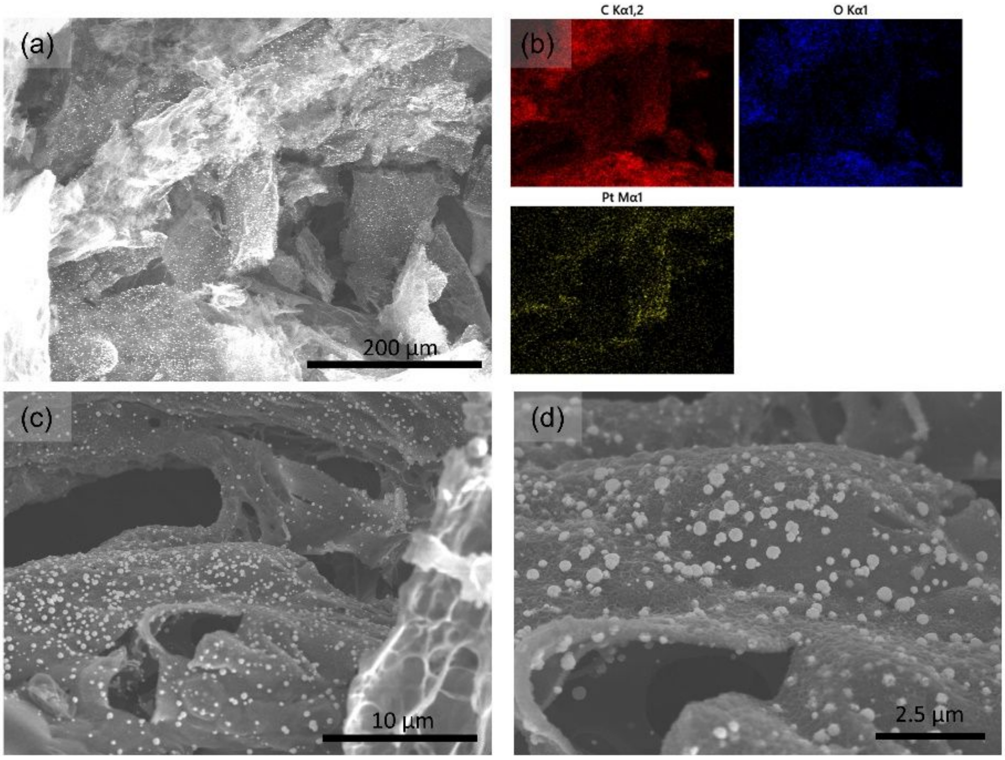

Figure S8 – SEM micrographs of electrodeposited PtNPs. (a) Far-off view of electrode surface, with a uniform distribution of particles at electrode surface. (b) EDS mapping of elemental distribution for C, O and Pt. (c and d) Magnified view of PtNPs distribution, showing a spherical geometry and sizes around 100 to 500 nm.

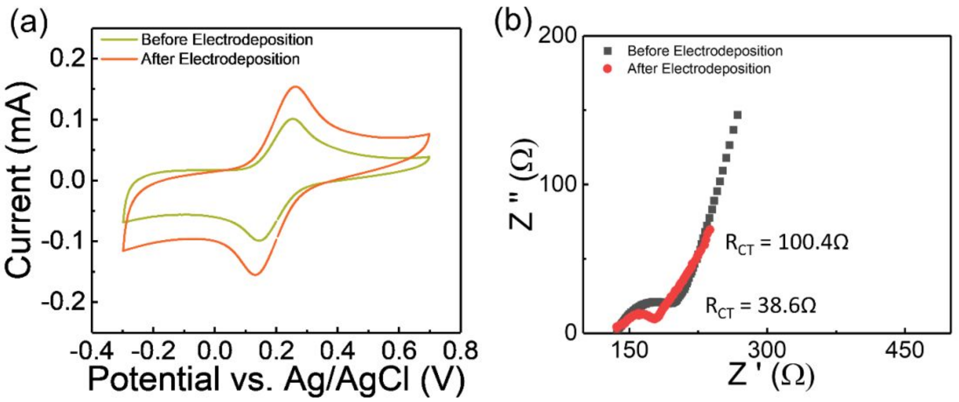

Figure S9 – Characterization of planar cells before and after PtNPs electrodeposition. (a) CVs at 50 mV.s^{-1}, showing an increase in peak currents for electrodeposition at the WE. (b) Nyquist plots showing decreased charge transfer resistances.

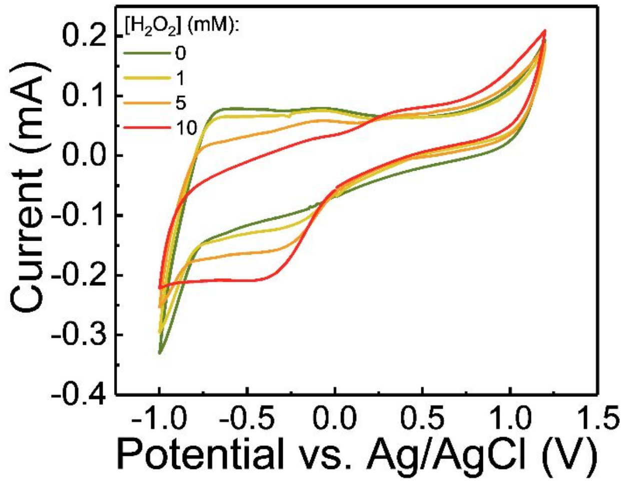

Figure S10 – CVs of the response of PtNPs modified WE in the presence of different concentrations of H_2O_2 in PBS buffer at 50 mV.s^{-1}. Both anodic currents at positive potential biases and cathodic current at more negative potential biases are visible, due to both oxidation and reduction of H_2O_2 at platinum modified electrode surface. Positive applied potentials were selected for analysis of sensor performance.

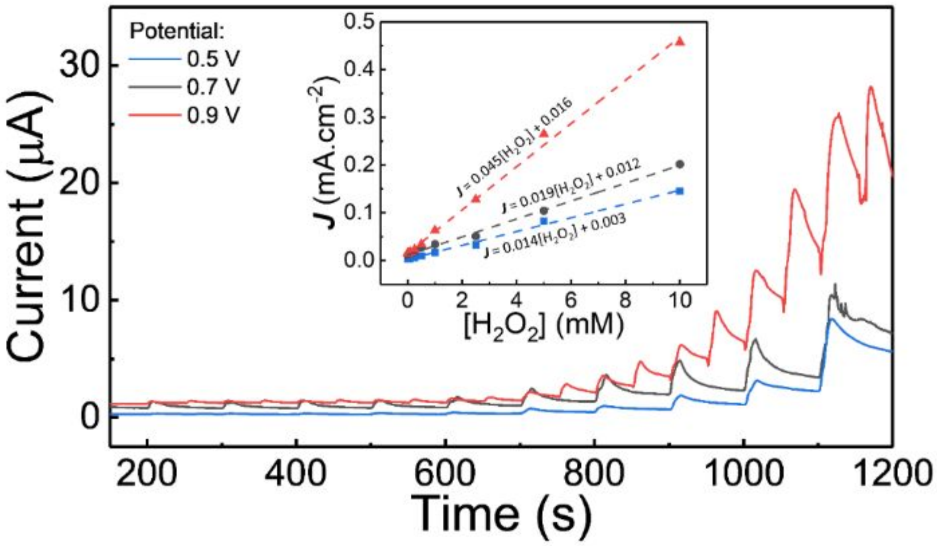

Figure S11 – Chronoamperometry study of sensor response to increasing concentration of H_2O_2 at different applied potentials (0.5 to 0.9 V), showing variation in sensitivity.

Table S2 – Comparison of LIG-based electrochemical hydrogen peroxide sensors.

Material	Linear range (µM)	Sensitivity (µA.mM^{-1}.cm^{-2})	LOD (µM)	Ref
PI-LIG/Au/PtNPs	10-3760	69.33	2.2	[16]
PI-LIG/sputtered PtNPs	0.5-5000	248.4	0.1	[17]
PI-LIG/Cu-Ru NPs	10-4320	136.7	1.8	[18]
PI-LIG/PB in electropolymerized PEDOT	10-1760	24.33	2.5	[19]
PI-LIG/ZnO	0.8-14600	276.8	190	[20]
PI-LIG/MWCNT	2000-12000	6.25	----	[21]
PI-LIG/PB	1-200	0.1	0.26	[22]
PI-LIF/AgNPs	10-550	28.6	2.8	[23]
Wax modified Paper-LIG/PtNPs	50-13200	16	11	This work

PtNPs – platinum nanoparticles
MWCNT – multiwalled carbon nanotube
PB – prussian Blue
PEDOT - Poly(3,4-ethylenedioxythiophene)
ZnO – zinc oxide
AgNPs – silver nanoparticles

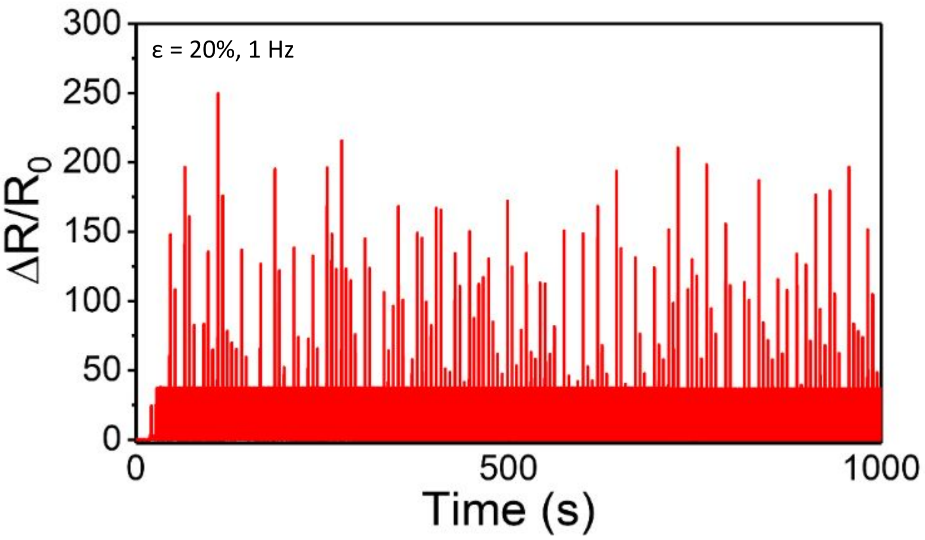

Figure S12 – Response of flexible strain gauges to bending strain cycling at 20% strain and 1 Hz frequency, showing the presence of triboelectric effects attributed to the polyurethane transfer substrate, as seen by the spikes in $\Delta R/R_0$.

Table S3 – Comparison of LIG-based strain sensor performance.

Material	Laser Source	Strain/Dynamic range	GF	Ref
PI-LIG	IR, CO_2	1.4 %	11.2	[24]
PI-LIG	UV, Nd:YVO_4	1%	26	[25]
PI-LIG transferred to PDMS	IR, CO_2	Up to 100%	20000	[26]
PI-LIG transferred to PU	IR, CO_2	Up to 100%	70	[27]
PI-LIG transferred to silicone rubber	IR, CO_2	31.8 %	37.8	[28]
Lignin embedded PDMS-LIG	UV, 355 nm	70 %	20	[29]
Lignin-LIG transferred to elastomeric Dragonskin™	IR, CO_2	14 %	960	[30]
Kevlar textile-LIG	Femtosecond YB fiber laser, 1030 nm	7 %	185.2	[31]
Paper-LIG	IR, CO_2	1.67 %	41.9	[12]
Paper-LIG transferred to PU	IR, CO_2	20 %	128.9	This work

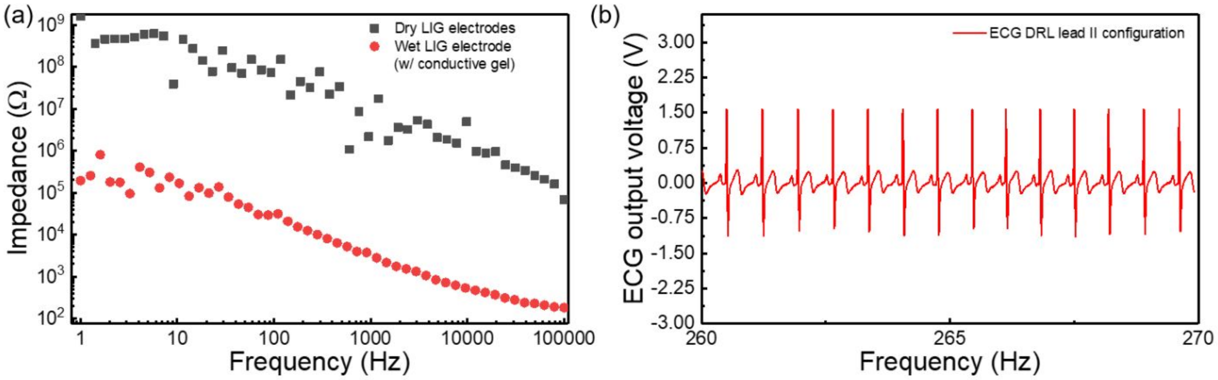

Figure S13 – Application of paper-based LIG transferred to PU for electrophysiological signal acquisition. (a) Electrode impedance measured at the forearm (3 cm distance, 5 mm diameter electrodes)) for dry and wet electrode configurations. (b) ECG signal for DRL lead II configuration using three LIG electrodes.

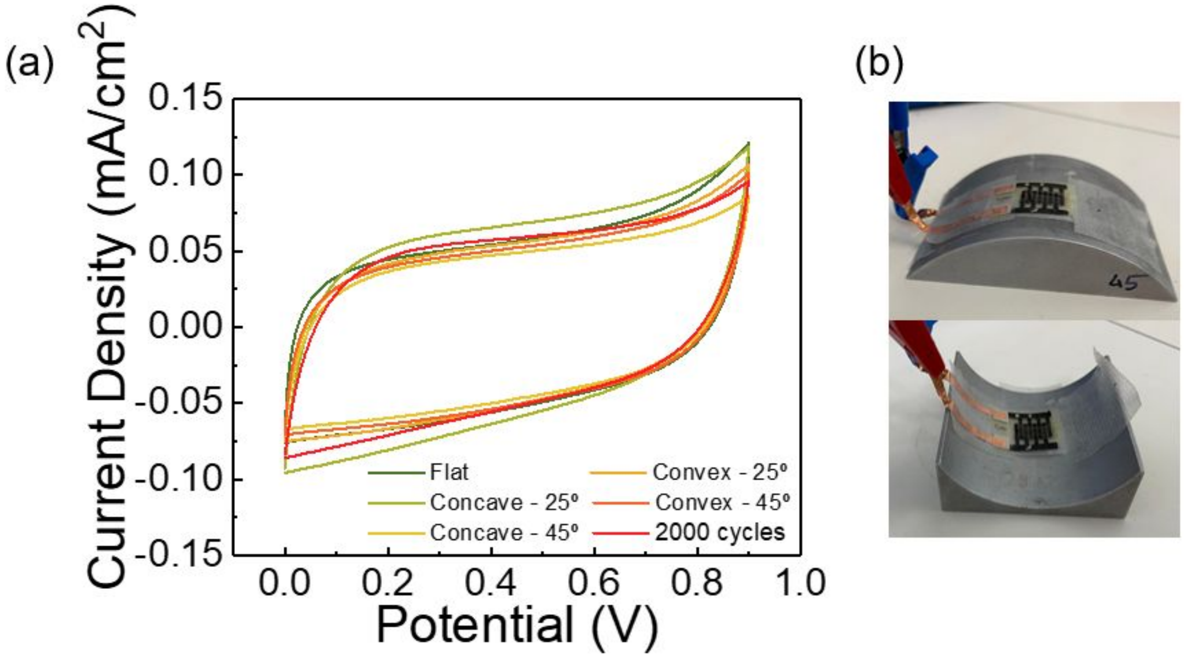

Figure S14 – Characterization of MSCs at (a) different bending angles using CV at 10 mV.s^{-1}. (b) Image of electrochemical cells at concave and convex testing angles.

Table S4 - Comparison of LIG-based microsupercapacitors performance.

Material	Laser Source	Electrolyte	Capacitance	Capacitance retention	Ref.
PI-LIG	IR, CO_2	Aqueous H_2SO_4	5 mF.cm^{-2} @ 20 mV.s^{-1}	93.7 % (7000 cycles @ 100 mV.s^{-1})	1
PI-LIG	Visible (450 nm)	PVA/H_3PO_4	0.62 mF.cm^{-2} @ 5 mV.s^{-1}	103 % (10000 cycles @ 0.11 mA.cm^{-2})	32
PI-LIG	IR, CO_2	PVA/H_3PO_4	55.5 mF.cm^{-2} @ 0.05 mA.cm^{-2}	Over 85 % (>10000 cycles @ 100 mV.s^{-1})	33
PI-LIG/NiO/CO_3O_4 transferred to WPU	IR, CO_2	PVA/H_3PO_4	2.4 mF.cm^{-2} @ 0.05 mA.cm^{-2}	98.4 % (10000 cycles @ 0.05 mA.cm^{-2})	34
PI-LIG transferred to adhesive textile	IR, CO_2	PVA/H_3PO_4	0.76 mF.cm^{-2} @ 3.6 μA cm^{-2}	93.6 % (5000 cycles @ 0.01 mA.cm^{-2})	35
Wood-LIG	Femtosecond UV (343 nm)	Na_2SO_4	3.5 mF.cm^{-2} @ 1 mA cm^{-2}	----	7
Cork-LIG	Visible (450 nm)	PVA-H_2SO_4	1.56 mF.cm^{-2} @ 0.1 mA cm^{-2}	99.7 % (5000 cycles @ 0.1 mA cm^{-2})	36
Kraft lignin-LIG	IR, CO_2	PVA-H_2SO_4	0.88 mF.cm^{-2} @ 10 mV.s^{-1}	91 % (10000 cycles @ 0.02 mA cm^{-2})	37
PES/Lignin-LIG	IR, CO_2	PVA-H_2SO_4	22 mF.cm^{-2} @ 0.05 mA cm^{-2}	89.8 % (9000 cycles @ 0.5 mA cm^{-2})	38
Paper-LIG transferred to PU	IR, CO_2	PVA-H_2SO_4	3.77 mF.cm^{-2} @ 0.1 mA cm^{-2}	95 % (10000 cycles @ 0.5 mA cm^{-2})	This work

References:

(1) Lin, J.; Peng, Z.; Liu, Y.; Ruiz-Zepeda, F.; Ye, R.; Samuel, E. L. G.; Yacaman, M. J.; Yakobson, B. I.; Tour, J. M. Laser-Induced Porous Graphene Films from Commercial Polymers. *Nat. Commun.* **2014**, *5* (1), 5714.

(2) Meng, L.; Turner, A. P. F.; Mak, W. C. Conducting Polymer-Reinforced Laser-Irradiated Graphene as a Heterostructured 3D Transducer for Flexible Skin Patch Biosensors. *ACS Appl. Mater. Interfaces* **2021**, *13* (45), 54456–54465.

(3) Chyan, Y.; Ye, R.; Li, Y.; Singh, S. P.; Arnusch, C. J.; Tour, J. M. Laser-Induced Graphene by Multiple Lasing: Toward Electronics on Cloth, Paper, and Food. *ACS Nano* **2018**, *12* (3), 2176–2183.

(4) Zhang, Z.; Song, M.; Hao, J.; Wu, K.; Li, C.; Hu, C. Visible Light Laser-Induced Graphene from Phenolic Resin: A New Approach for Directly Writing Graphene-Based Electrochemical Devices on Various Substrates. *Carbon N. Y.* **2018**, *127*, 287–296.

(5) Wang, G.; Tao, L. Q.; Peng, Z.; Zhu, C.; Sun, H.; Zou, S.; Li, T.; Wang, P.; Chen, X.; Ren, T. L. Nomex Paper-Based Double-Sided Laser-Induced Graphene for Multifunctional Human-Machine Interfaces. *Carbon N. Y.* **2022**, *193*, 68–76.

(6) Ye, R.; Chyan, Y.; Zhang, J.; Li, Y.; Han, X.; Kittrell, C.; Tour, J. M. Laser-Induced Graphene Formation on Wood. *Adv. Mater.* **2017**, *29* (37), 1702211.

(7) Le, T. S. D.; Park, S.; An, J.; Lee, P. S.; Kim, Y. J. Ultrafast Laser Pulses Enable One-Step Graphene Patterning on Woods and Leaves for Green Electronics. *Adv. Funct. Mater.* **2019**, *29* (33), 1902771.

(8) Dreimol, C. H.; Guo, H.; Ritter, M.; Keplinger, T.; Ding, Y.; Günther, R.; Poloni, E.; Burgert, I.; Panzarasa, G. Sustainable Wood Electronics by Iron-Catalyzed Laser-Induced Graphitization for Large-Scale Applications. *Nat. Commun. 2022 131* **2022**, *13* (1), 1–12.

(9) Le, T. D.; Lee, Y. A.; Nam, H. K.; Jang, K. Y.; Yang, D.; Kim, B.; Yim, K.; Kim, S.; Yoon, H.; Kim, Y. Green Flexible Graphene–Inorganic-Hybrid Micro-Supercapacitors Made of Fallen Leaves Enabled by Ultrafast Laser Pulses. *Adv. Funct. Mater.* **2021**, 2107768.

(10) Edberg, J.; Brooke, R.; Hosseinaei, O.; Fall, A.; Wijeratne, K.; Sandberg, M. Laser-Induced Graphitization of a Forest-Based Ink for Use in Flexible and Printed Electronics. *npj Flex. Electron.* **2020**, *4* (1), 1–10.

(11) Park, H.; Kim, M.; Kim, B. G.; Kim, Y. H. Electronic Functionality Encoded Laser-Induced Graphene for Paper Electronics. *ACS Appl. Nano Mater.* **2020**, *3* (7), 6899–6904.

(12) Kulyk, B.; Silva, B. F. R.; Carvalho, A. F.; Silvestre, S.; Fernandes, A. J. S.; Martins, R.; Fortunato, E.; Costa, F. M. Laser-Induced Graphene from Paper for Mechanical Sensing. *ACS Appl. Mater. Interfaces* **2021**, *13* (8), 10210–10221.

(13) Pinheiro, T.; Silvestre, S.; Coelho, J.; Marques, A. C.; Martins, R.; Sales, M. G. F.; Fortunato, E. Laser-Induced Graphene on Paper toward Efficient Fabrication of Flexible, Planar Electrodes for Electrochemical Sensing. *Adv. Mater. Interfaces* **2021**, *8* (22), 2101502.

(14) Kulyk, B.; Matos, M.; Silva, B. F. R.; Carvalho, A. F.; Fernandes, A. J. S.; Evtuguin, D. V.; Fortunato, E.; Costa, F. M. Conversion of Paper and Xylan into Laser-Induced Graphene for Environmentally Friendly Sensors. *Diam. Relat. Mater.* **2022**, *123*, 108855.

(15) Kulyk, B.; Silva, B. F. R.; Carvalho, A. F.; Barbosa, P.; Girão, A. V.; Deuermeier, J.; Fernandes, A. J. S.; Figueiredo, F. M. L.; Fortunato, E.; Costa, F. M. Laser-Induced Graphene from Paper by Ultraviolet Irradiation: Humidity and Temperature Sensors. *Adv. Mater. Technol.* **2022**, 2101311.

(16) Yoon, H.; Nah, J.; Kim, J.; Xuan, X.; Park, J. Laser-Induced Graphene Stamp for High Performacne Elecrochemical Sensing Applications. *Proc. IEEE Int. Conf. Micro Electro Mech. Syst.* **2019**, *2019-January*, 537–540.

(17) Zhang, Y.; Zhu, H.; Sun, P.; Sun, C. K.; Huang, H.; Guan, S.; Liu, H.; Zhang, H.; Zhang, C.; Qin, K. R. Laser-Induced Graphene-Based Non-Enzymatic Sensor for Detection of Hydrogen Peroxide. *Electroanalysis* **2019**, *31* (7), 1334–1341.

(18) Thirumalai, D.; Lee, J.-U.; Choi, H.; Kim, M.; Lee, J.; Kim, S.; Shin, B.-S.; Chang, S.-C. In Situ Synthesis of Copper-Ruthenium Bimetallic Nanoparticles on Laser-Induced Graphene as a Peroxidase Mimic †.

(19) Meng, L.; Turner, A. P. F.; Mak, W. C. Conducting Polymer-Reinforced Laser-Irradiated Graphene as a Heterostructured 3D Transducer for Flexible Skin Patch Biosensors. *Cite This ACS Appl. Mater. Interfaces* **2021**, *13*, 54465.

(20) Zanoni, J.; Moura, J. P.; Santos, N. F.; Carvalho, A. F.; Fernandes, A. J. S.; Monteiro, T.; Costa, F. M.; Pereira, S. O.; Rodrigues, J. Dual Transduction of H2o2 Detection Using Zno/Laser-Induced Graphene Composites. *Chemosensors* **2021**, *9* (5), 102.

(21) Settu, K.; Lai, Y. C.; Liao, C. T. Carbon Nanotube Modified Laser-Induced Graphene Electrode for Hydrogen Peroxide Sensing. *Mater. Lett.* **2021**, *300*, 130106.

(22) Matias, T. A.; de Faria, L. V.; Rocha, R. G.; Silva, M. N. T.; Nossol, E.; Richter, E. M.; Muñoz, R. A. A. Prussian Blue-Modified Laser-Induced Graphene Platforms for Detection of Hydrogen Peroxide. *Microchim. Acta* **2022**, *189* (5), 1–9.

(23) Zhao, G.; Wang, F.; Zhang, Y.; Su, Y.; Liu, P.; Zhan, Z.; Xu, C.; Yang, C. High-Performance Hydrogen Peroxide Micro-Sensors Based on Laser-Induced Fabrication of Graphene@Ag Electrodes. *Appl. Surf. Sci.* **2021**, *565*, 150565.

(24) Kaidarova, A.; Khan, M. A.; Marengo, M.; Swanepoel, L.; Przybysz, A.; Muller, C.; Fahlman, A.; Buttner, U.; Geraldi, N. R.; Wilson, R. P.; Duarte, C. M.; Kosel, J. Wearable Multifunctional Printed Graphene Sensors. *npj Flex. Electron. 2019 31* **2019**, *3* (1), 1–10.

(25) Carvalho, A. F.; Fernandes, A. J. S.; Leitão, C.; Deuermeier, J.; Marques, A. C.; Martins, R.; Fortunato, E.; Costa, F. M. Laser-Induced Graphene Strain Sensors Produced by Ultraviolet Irradiation of Polyimide. *Adv. Funct. Mater.* **2018**, *28* (52), 1805271.

(26) Rahimi, R.; Ochoa, M.; Yu, W.; Ziaie, B. Highly Stretchable and Sensitive Unidirectional Strain Sensor via Laser Carbonization. *ACS Appl. Mater. Interfaces* **2015**, *7* (8), 4463–4470.

(27) Dallinger, A.; Keller, K.; Fitzek, H.; Greco, F. Stretchable and Skin-Conformable Conductors Based on Polyurethane/Laser-Induced Graphene. *ACS Appl. Mater. Interfaces* **2020**, *12* (17), 19855–19865.

(28) Huang, L.; Wang, H.; Wu, P.; Huang, W.; Gao, W.; Fang, F.; Cai, N.; Chen, R.; Zhu, Z. Wearable Flexible Strain Sensor Based on Three-Dimensional Wavy Laser-Induced Graphene and Silicone Rubber. *Sensors 2020, Vol. 20, Page 4266* **2020**, *20* (15), 4266.

(29) Lee, C. W.; Jeong, S. Y.; Kwon, Y. W.; Lee, J. U.; Cho, S. C.; Shin, B. S. Fabrication of Laser-Induced Graphene-Based Multifunctional Sensing Platform for Sweat Ion and Human Motion Monitoring. *Sensors Actuators A Phys.* **2022**, *334*, 113320.

(30) Yang, S.; Ling, Y.; Wu, Q.; Zhang, H.; Yan, Z.; Huang, G.; Lin, J.; Wan, C. Lignin-Derived Porous Graphene for Wearable and Ultrasensitive Strain Sensors. *J. Mater. Chem. C* **2022**, *10* (32), 11730–11738.

(31) Kim, Y. J.; Yang, D.; Nam, H. K.; Le, T. S. D.; Lee, Y.; Kwon, S. Direct-Laser-Conversion of Kevlar Textile to Laser-Induced-Graphene for Realizing Fast and Flexible Fabric Strain

Sensors. *CIRP Ann.* **2022**, *71* (1), 473–476.

(32) Shi, X.; Zhou, F.; Peng, J.; Wu, an; Wu, Z.-S.; Bao, X.; Shi, X. Y.; Bao, X. H.; Zhou, F.; Wu, Z.; Peng, J. X.; Wu, R. A. One-Step Scalable Fabrication of Graphene-Integrated Micro-Supercapacitors with Remarkable Flexibility and Exceptional Performance Uniformity. *Adv. Funct. Mater.* **2019**, *29* (50), 1902860.

(33) Rao, Y.; Yuan, M.; Luo, F.; Wang, Z.; Li, H.; Yu, J.; Chen, X. One-Step Laser Fabrication of Phosphorus-Doped Porous Graphene Electrodes for High-Performance Flexible Microsupercapacitor. *Carbon N. Y.* **2021**, *180*, 56–66.

(34) Wang, W.; Lu, L.; Xie, Y.; Yuan, W.; Wan, Z.; Tang, Y.; Teh, K. S. A Highly Stretchable Microsupercapacitor Using Laser-Induced Graphene/NiO/Co3O4 Electrodes on a Biodegradable Waterborne Polyurethane Substrate. *Adv. Mater. Technol.* **2020**, *5* (2), 1900903.

(35) Kwon, S.; Lee, T.; Choi, H. J.; Ahn, J.; Lim, H.; Kim, G.; Choi, K. B.; Lee, J. J. Scalable Fabrication of Inkless, Transfer-Printed Graphene-Based Textile Microsupercapacitors with High Rate Capabilities. *J. Power Sources* **2021**, *481*, 228939.

(36) Imbrogno, A.; Islam, J.; Santillo, C.; Castaldo, R.; Sygellou, L.; Larrigy, C.; Murray, R.; Vaughan, E.; Hoque, M. K.; Quinn, A. J.; Iacopino, D. Laser-Induced Graphene Supercapacitors by Direct Laser Writing of Cork Natural Substrates. *ACS Appl. Electron. Mater.* **2022**, *4* (4), 1541–1551.

(37) Mahmood, F.; Mahmood, F.; Zhang, H.; Lin, J.; Wan, C. Laser-Induced Graphene Derived from Kraft Lignin for Flexible Supercapacitors. *ACS Omega* **2020**, *5* (24), 14611–14618.

(38) Sun, X.; Liu, X.; Li, F. Sulfur-Doped Laser-Induced Graphene Derived from Polyethersulfone and Lignin Hybrid for All-Solid-State Supercapacitor. *Appl. Surf. Sci.* **2021**, *551*, 149438.
